# Delays to revascularization for patients with chronic limb-threatening ischaemia

**DOI:** 10.1093/bjs/znac109

**Published:** 2022-05-11

**Authors:** Qiuju Li, Panagiota Birmpili, Amundeep S Johal, Sam Waton, Arun D Pherwani, Jonathan R Boyle, David A Cromwell

**Affiliations:** Department of Health Services Research and Policy, London School of Hygiene and Tropical Medicine, London, UK; Clinical Effectiveness Unit, Royal College of Surgeons of England, London, UK; Clinical Effectiveness Unit, Royal College of Surgeons of England, London, UK; Hull York Medical School, Hull, UK; Clinical Effectiveness Unit, Royal College of Surgeons of England, London, UK; Clinical Effectiveness Unit, Royal College of Surgeons of England, London, UK; Vascular Surgery, Royal Stoke University Hospital, University Hospitals of North Midlands NHS Trust, Stoke-on-Trent, UK; Cambridge Vascular Unit, Cambridge University Hospitals NHS Foundation Trust & Department of Surgery, University of Cambridge, Cambridge, UK; Department of Health Services Research and Policy, London School of Hygiene and Tropical Medicine, London, UK; Clinical Effectiveness Unit, Royal College of Surgeons of England, London, UK

## Abstract

**Background:**

Vascular services in England are organized into regional hub-and-spoke models, with hubs performing arterial surgery. This study examined time to revascularization for chronic limb-threatening ischaemia (CLTI) within and across different care pathways, and its association with postrevascularization outcomes.

**Methods:**

Three inpatient and four outpatient care pathways were identified for patients with CLTI undergoing revascularization between April 2015 and March 2019 using Hospital Episode Statistics data. Differences in times from presentation to revascularization across care pathways were analysed using Cox regression. The relationship between postoperative outcomes and time to revascularization was evaluated by logistic regression.

**Results:**

Among 16 483 patients with CLTI, 9470 had pathways starting with admission to a hub or spoke hospital, whereas 7013 (42.5 per cent) were first seen at outpatient visits. Among the inpatient pathways, patients admitted to arterial hubs had shorter times to revascularization than those admitted to spoke hospitals (median 5 (i.q.r. 2–10) *versus* 12 (7–19) days; *P* < 0.001). Shorter times to revascularization were also observed for patients presenting to outpatient clinics at arterial hubs compared with spoke hospitals (13 (6–25) *versus* 26 (15–35) days; *P* < 0.001). Within most care pathways, longer delays to revascularizsation were associated with increased risks of postoperative major amputation and in-hospital death, but the effect of delay differed across pathways.

**Conclusion:**

For patients with CLTI, time to revascularization was influenced by presentation to an arterial hub or spoke hospital. Generally, longer delays to revascularization were associated with worse outcomes, but the impact of delay differed across pathways.

## Teaser

This study examined time to revascularization from presentation for patients with chronic limb-threatening ischaemia (CLTI) within and across different care pathways within the reginal hub-and-spoke models of vascular service networks, using Hospital Episode Statistics data between April 2015 and March 2019. The data suggested that, among the patients with CLTI, time to revascularization was influenced by presentation to an arterial hub or spoke hospital. This study also suggested that, generally, longer delays to revascularization were associated with worse postrevascularization outcomes of major amputation and in-hospital death, but the impact of delay differed across care pathways.

## Introduction

Chronic limb-threatening ischaemia (CLTI) is a severe form of peripheral arterial disease (PAD) in the lower limbs characterized by rest pain and/or tissue loss, such as ulceration or gangrene (Fontaine classification III or IV)^1,2^. The symptoms result from reduced blood flow in the legs, and revascularization is required to improve blood flow and reduce the risk of limb loss. Revascularization may be performed using either endovascular techniques (angioplasty and/or stenting), open surgery (lower limb bypass procedures, endarterectomy) or a hybrid combination of procedures, depending on the patient’s risk, severity of limb threatening ischaemia, and anatomical patterns of disease^1–4^.

Centralization has been a common strategy for highly specialized surgery in healthcare systems in Europe and North America^5,6^. In response to the evidence that greater surgeon and vascular unit volumes improve patient outcomes^7–9^, there has been a centralization of vascular arterial surgical services in the National Health Service (NHS)^10^, with a hub-and-spoke model^11^ introduced within geographical regions. In these regional vascular networks, arterial hubs provide arterial surgery and complex endovascular interventions. Non-arterial spoke hospitals provide outpatient services including local assessment and diagnostic services, and, where appropriate, day-case peripheral angioplasty and stenting^10^. Patients admitted to a non-arterial spoke hospital are transferred to the regional vascular arterial hub when they require an operative procedure^10,12^. Through reconfiguration, the number of NHS acute Trusts that perform lower limb bypass operations in England fell from 110 in 2011^13^ to 70 in 2017^14^.

There has been a long-standing concern that late presentation and delayed management of CLTI contributes to increased rates of lower limb amputation and mortality^15^. National guidance from the Vascular Society in the Provision of Vascular Services 2018 (POVS 2018)^10^ and the Peripheral Arterial Disease Quality Improvement Framework (PAD QIF) in 2019^16^ recommends revascularization within 5 days of hospital admission for patients with severe CLTI, or within 14 days of outpatient referral for those who present with stable disease. To ensure patients have rapid access to both endovascular and surgical revascularization, vascular networks need to have effective referral pathways. Failure to achieve this could result in extended delays, particularly for patients who first present at a spoke hospital before having revascularization at an arterial centre. The POVS 2018 guidance^10^ states that ‘equal access to treatment should occur irrespective of where in the network a patient presents’. Therefore, the aim of this study was to examine how time to revascularization for patients with CLTI might vary depending on their care pathway across NHS hospitals in England. This study also investigated the impact of time to revascularization on adverse short-term outcomes, including in-hospital mortality and the risk of subsequent major lower limb amputation during the same admission following revascularization.

## Methods

The study used a data set extracted from the inpatient Hospital Episode Statistics (HES) database held by NHS England. The inpatient database codes diagnostic information using ICD-10, and operative procedures using OPCS-4. The study cohort consisted of patients aged 35 years and over, admitted as an emergency with CLTI-related diagnostic codes (*Table S1*) to a NHS hospital in England for a lower limb revascularization procedure (codes listed in *Table S2*) between 1 April 2015 and 31 March 2019. Because patients who had previous revascularization procedures may have followed different care pathways with potentially more adverse outcomes, only the first revascularization procedure for each patient was included. Patients were excluded if they had other lower limb revascularization procedures recorded within 2 years before the start of the study interval (1 April 2015). The OPCS procedure codes were used to distinguish between endovascular (angioplasty/stent), open (bypass/endarterectomy), and hybrid procedures. A hybrid procedure was recorded where both endovascular and open surgical operations were performed on the same date. Patients with end-stage renal disease and on dialysis were excluded, because special care might be required to accommodate their dialysis requirements and potentially prolong waiting times to revascularization. Patients who had both revascularization and major amputation on the date of the first lower limb procedure were defined as those undergoing a primary amputation and also excluded. The analysis was restricted to NHS hospitals in England which had not changed their status from an arterial centre (hub) to a non-arterial centre (spoke) during the study interval as a result of reconfiguration of vascular services, and to patients who resided in England at the time of revascularization.

### Care pathway definitions

Within the hub-and-spoke network model for vascular services in the UK, patients with CLTI could either be admitted directly to a hospital as an emergency (inpatient pathway), or referred to an outpatient clinic for specialist assessment before a treatment decision was made (outpatient pathway). The first contact with vascular services preceding revascularization was identified using patient records from both inpatient and outpatient HES data sets. When patients had an outpatient visit with a specialist in vascular surgery, diabetic medicine, podiatry or general surgery within 30 days before admission for revascularization, they were classed as following outpatient care pathways. This definition was used owing to the multidisciplinary nature of foot care, and because some vascular surgeons were still coded as performing as specialists in general surgery. The earliest outpatient visit was defined as the first contact with vascular services. Patients were classed as following inpatient care pathways if they had no vascular-related outpatient visits preceding revascularization and were admitted non-electively. This included patients who (following the initial admission for CLTI) were transferred to another hospital, and/or discharged and then readmitted for revascularization. The interval between discharge and readmission was limited to 30 days.

There was a total of 19 distinct pathways starting with either an admission or outpatient visit to an arterial vascular hub or a non-arterial spoke hospital (*Table S3*). These were collated to form seven pathways that captured the type of first contact (inpatient or outpatient), whether that contact was at a hospital with an arterial hub, and whether or not the patient was discharged from hospital before revascularization. Patients starting with an admission were grouped into three inpatient care pathways, whereas those who were initially seen at an outpatient clinic were grouped into four outpatient care pathways (*Table 1*). To reduce heterogeneity in the overall cohort and focus on the most common pathways in current clinical practice, a small number of patients were excluded from these seven categories: those who initially presented to an arterial hub hospital and subsequently underwent revascularization at a different arterial centre, and patients who presented to an arterial hub hospital and had a subsequent endovascular procedure at a non-arterial spoke hospital.

**Table 1:** Description of care pathways to revascularization in English National Health Service

	Label	Description
**Inpatient pathway—first contact: emergency admission**		
1	Adm(Hub)	Admission to an arterial hub hospital and revascularization during the same admission
2	Adm(Spoke/transfer)	Admission to a non-arterial spoke hospital and revascularization at the same spoke unit or transfer to a hub or another spoke unit for revascularization
3	Adm(Any)-Dis+Readm	Admission to a spoke or hub unit, subsequent discharge and readmission to a spoke or hub for revascularization
**Outpatient pathway—first contact: outpatient visit**		
4	OP(Hub)-Adm(Hub)	Outpatient visit at an arterial hub hospital and admission to the hub unit for revascularization
5	OP(Spoke)-Adm(Hub)	Outpatient visit at a non-arterial spoke hospital and admission to a hub unit for revascularization
6	OP(Spoke)-Adm(Spoke)	Outpatient visit at a non-arterial spoke hospital and admission to spoke unit for revascularization or admission to spoke unit then transfer to hub unit for revascularization
7	OP-Adm-Dis+Readm	Outpatient visit at an arterial hub or a non-arterial spoke hospital and admission, followed by discharge and readmission to either a spoke or hub for revascularization

Adm, admission; Dis, discharge; Readm, readmission; OP, outpatient.

### Outcome and explanatory variables

The primary outcome was time to revascularization from the point of first contact (outpatient visit or inpatient admission, as appropriate). The study adopted the POVS 2018^10^/PAD QIF^16^ standards on time to revascularization: 5 days from a non-elective admission and 14 days from an outpatient visit. The proportion of patients whose time to revascularization exceeded 5 days following inpatient pathways or 14 days following outpatient pathways was calculated for each care pathway. Secondary outcomes were the proportion of patients undergoing major lower limb amputation after revascularization during the same admission, and the proportion of patients who died in hospital after revascularization. The outcome variable for major amputation included all procedures, and did not distinguish between the sides of amputation and revascularization.

Patient characteristics taken from the admission episode were used for analyses. Patient demographics included age on admission, sex, and region of residence. Socioeconomic deprivation was measured using the English Index of Multiple Deprivation of a patient’s residential area and converted to quintiles based on a national ranking^17^. The severity of CLTI was categorized into two groups, depending on whether or not patients presented with tissue loss (ulceration, gangrene, and osteomyelitis).

Co-morbidities were captured using the Royal College of Surgeons (RCS) Charlson score^18^, which was derived using primary and secondary diagnostic codes from the index hospital admission (admission for revascularization) as well as admissions during the 12 months preceding the index admission. Acute conditions (such as myocardial infarction) were included in the number of co-morbidities only if they were present in a record of a hospital admission preceding the index admission. Diagnostic codes for PAD and diabetes were excluded from the RCS Charlson co-morbidity score in this study. The PAD codes formed part of the inclusion criteria for the study, whereas diabetes status was examined as a separate variable.

### Statistical analysis

Descriptive statistics were used to describe the demographic and clinical characteristics of the patient cohort. The distribution of time to revascularization for each care pathway was summarized using the median and quartiles, and presented graphically in a box plot. Differences in time to revascularization between patients following different care pathways were examined using the Mann–Whitney *U* test. Cox regression was used to assess the association between time to revascularization and patient and clinical characteristics. The NHS Trusts performing revascularization were included as random effects in the multivariable Cox regression models to account for similarities in vascular services received among patients in the same Trust compared with the whole population. A half day was applied to time to revascularization in the Cox regression models where the first contact was on the same date as revascularization. A hazard ratio of less than 1 for a subgroup indicated that the time to revascularization tended to be longer for patients in that subgroup compared with patients in the reference group.

An initial exploration of the relationship between time to revascularization and the short-term risks of postoperative mortality and major amputation during the same admission was performed visually using a symmetric nearest-neighbour smoother^19,20^. A multivariable logistic regression model was used to assess their associations with time to revascularization, adjusting for other covariates of interest. Linear and quadratic terms of log transformation of time to revascularization were explored in the models. The NHS Trusts were included as random effects in the models to account for similarities in the postoperative outcomes among patients treated in the same organization compared with the whole population.

Separate regression analyses were carried out for patients following inpatient and outpatient care pathways. Owing to the possibly inaccurate clinical coding in administrative hospital data, sensitivity analyses that included additional patients with a primary diagnostic code for acute limb ischaemia and secondary diagnostic codes for CLTI were undertaken for all Cox and logistic regression analyses conducted in the main analysis. Sensitivity analyses were also conducted to explore the impact of outpatient specialties, and the time limit between the outpatient visit and the admission for revascularization. The analyses involved using a 15-day limit, a 60-day limit, and specialist in vascular surgery only. All statistical tests were two-sided and *P* < 0.050 was considered statistically significant. All analyses were done using Stata^®^ MP version 15 (StataCorp, College Station, Texas, USA).

## Results

The study identified 23 274 patients aged at least 35 years who underwent lower limb procedures with an emergency admission for CLTI between April 2015 and March 2019. Of these, 17 623 (75.7 per cent) underwent revascularization as the first lower limb procedure, whereas 5651 (24.3 per cent) underwent primary amputation and were excluded. Of those undergoing revascularization, the following were excluded: 404 patients (2.3 per cent) who were on dialysis at the time of revascularization; 458 (2.6 per cent) treated at eight hospitals that had changed status from an arterial hub to a non-arterial spoke site; 142 (0.8 per cent) whose first contact for CLTI was at an arterial hub, but who subsequently underwent revascularization elsewhere; and 136 (0.8 per cent) whose time to revascularization exceeded 70 days. These exclusions left 16 483 patients for analysis.

### Patient characteristics

Characteristics of patients included in the analyses are summarized in *Table 2*. The majority were men (65.3 per cent), and aged 70 years and over (62.3 per cent). More than half of the patients (54.8 per cent) had diabetes, and two-thirds (67.1 per cent) had at least one other Charlson co-morbidity. At the time of revascularization, 59.0 per cent of patients had tissue loss. Overall, 9470 patients (57.5 per cent) followed care pathways that started with a hospital admission, whereas 7013 (42.5 per cent) followed care pathways that started with an outpatient visit. Among those who followed an outpatient pathway, 60.4 per cent had diabetes, whereas 50.6 per cent of those who followed an inpatient pathway were diabetic. A slightly higher proportion of patients had tissue loss at the time of revascularization among those who followed an outpatient pathway than those who followed an inpatient pathway (62.4 *versus* 56.6 per cent respectively). The proportion of patients who followed each care pathway varied across English regions (*Fig. S1*).

**Table 2:** Characteristics of patients with chronic limb-threatening ischaemia at time of revascularization (between April 2015 and March 2019), stratified by type of first contact

	Inpatients (*n* = 9470)	Outpatients (*n* = 7013)	Total (*n* = 16 483)
**Men**	6150 (64.9)	4619 (65.9)	10 769 (65.3)
**Age (years)**			
≤ 49	305 (3.2)	195 (2.8)	500 (3.0)
50–59	1053 (11.1)	837 (11.9)	1890 (11.5)
60–69	2189 (23.1)	1630 (23.2)	3819 (23.2)
70–79	2931 (31.0)	2217 (31.6)	5148 (31.2)
≥ 80	2992 (31.6)	2134 (30.4)	5126 (31.1)
**Deprivation quintile**			
Q1 (least deprived)	1317 (13.9)	1087 (15.5)	2404 (14.6)
Q2	1700 (18.0)	1239 (17.7)	2939 (17.8)
Q3	1855 (19.6)	1377 (19.6)	3232 (19.6)
Q4	2124 (22.4)	1471 (21.0)	3595 (21.8)
Q5 (most deprived)	2474 (26.1)	1839 (26.2)	4313 (26.2)
**Diabetes mellitus**	4791 (50.6)	4236 (60.4)	9027 (54.8)
**RCS Charlson score (diabetes not included)**			
0	3106 (32.8)	2315 (33.0)	5421 (32.9)
1	2883 (30.4)	2147 (30.6)	5030 (30.5)
2	1858 (19.6)	1390 (19.8)	3248 (19.7)
≥ 3	1623 (17.1)	1161 (16.6)	2784 (16.9)
**CLTI indicator**			
No record of tissue loss	4114 (43.4)	2640 (37.6)	6754 (41.0)
With record of tissue loss	5356 (56.6)	4373 (62.4)	9729 (59.0)
**Procedure**			
Endovascular	6644 (70.2)	5001 (71.3)	11 645 (70.6)
Open surgery	2235 (23.6)	1538 (21.9)	3773 (22.9)
Hybrid	591 (6.2)	474 (6.8)	1065 (6.5)
**Financial year**			
2015–2016	2465 (26.0)	1726 (24.6)	4191 (25.4)
2016–2017	2372 (25.1)	1719 (24.5)	4091 (24.8)
2017–2018	2430 (25.7)	1830 (26.1)	4260 (25.8)
2018–2019	2203 (23.3)	1738 (24.8)	3941 (23.9)

Values in parentheses are percentages. Financial year runs from 1 April to 31 March the following year. RCS, Royal College of Surgeons; CLTI, chronic limb-threatening ischaemia.

### Time to revascularization

**Fig. 1:**
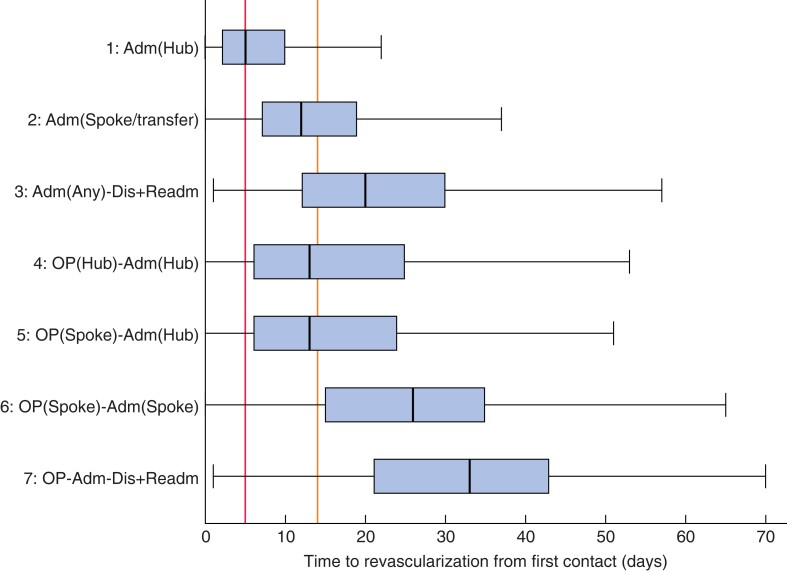
Time to revascularization from point of first contact with vascular services, by care pathway Median value (bold line), i.q.r. (box), and range excluding outliers (error bar) are shown. Outliers have not been plotted. The red line indicates 5 days and the orange line 14 days. Adm, admission; Dis, discharge; Readm, readmission; OP, outpatient.

The summary of time to revascularization for each care pathway is presented in *Tables 3* and *4*, and *Fig. 1* (data are shown for the 19 distinct pathways in *Fig. S2*). Of the seven pathways, patients admitted to an arterial hub hospital as an emergency admission (pathway 1) tended to have the shortest time to revascularization, with a median of 5 (i.q.r. 2–10) days among 5560 patients. The 1783 patients admitted to a non-arterial spoke hospital (pathway 2) had a median time of 12 (7–19) days, well in excess of the target 5 days and more than twice as long as that for patients admitted to an arterial hub hospital (*P* < 0.001). However, the 2127 patients who were discharged after the initial emergency admission (pathway 3) experienced even longer delays to revascularization, with a median of 20 (12–30) days, and only 7.0 per cent had a procedure within 5 days.

**Table 3:** Summary of lower limb revascularization procedures and postoperative outcomes, stratified by inpatient care pathway

	1: Adm(Hub) (*n* = 5560)	2: Adm(Spoke/transfer)(*n* = 1783)	3: Adm(Any)-Dis+Readm (*n* = 2127)
**Procedure**			
Endovascular	3797 (68.3)	1374 (77.1)	1473 (69.3)
Surgical	1409 (25.3)	314 (17.6)	512 (24.1)
Hybrid	354 (6.4)	95 (5.3)	142 (6.7)
**CLTI indicator**			
No record of tissue loss	2705 (48.7)	425 (23.8)	984 (46.3)
With record of tissue loss	2855 (51.3)	1358 (76.2)	1143 (53.7)
Time to revascularization (days)*	5 (2–10)	12 (7–19)	20 (12–30)
> 5 days after admission	2766 (49.7)	1447 (81.2)	1978 (93.0)
**Postoperative summary and outcomes**			
Duration of hospital stay (days)*	8 (3–18)	12 (5–25)	5 (1–14)
Major amputation	400 (7.2)	124 (7.0)	109 (5.1)
In-hospital death	336 (6.0)	112 (6.3)	109 (5.1)
Amputation-free survival at discharge	4877 (87.7)	1564 (87.7)	1917 (90.1)

Values in parentheses are percentages unless indicated otherwise; *values are median (i.q.r.). Adm, admission; Dis, discharge; Readm, readmission; CLTI, chronic limb-threatening ischaemia.

**Table 4:** Summary of lower limb revascularization procedures and postoperative outcomes, stratified by outpatient care pathway

	4: OP(Hub)-Adm(Hub) (*n* = 3530)	5: OP(Spoke)-Adm(Hub) (*n* = 1473)	6: OP(Spoke)-Adm(Spoke) (*n* = 634)	7: OP-Adm-Dis+Readm (*n* = 1376)
**Procedure**				
Endovascular	2536 (71.8)	1003 (68.1)	542 (85.5)	920 (66.9)
Surgical	767 (21.7)	352 (23.9)	74 (11.7)	345 (25.1)
Hybrid	227 (6.4)	118 (8.0)	18 (2.8)	111 (8.1)
**CLTI indicator**				
No record of tissue loss	1321 (37.4)	592 (40.2)	137 (21.6)	590 (42.9)
With record of tissue loss	2209 (62.6)	881 (59.8)	497 (78.4)	786 (57.1)
Time to revascularization (days)*	13 (6–25)	13 (6–24)	26 (15–35)	33 (21–43)
> 14 days after outpatient visit	1585 (44.9)	668 (45.3)	483 (76.2)	1175 (85.4)
**Postoperative summary and outcomes**				
Duration of hospital stay (days)*	7 (3–15)	7 (3–15)	8 (3–18)	4 (1–12)
Major amputation	203 (5.8)	87 (5.9)	35 (5.5)	60 (4.4)
In-hospital death	164 (4.6)	64 (4.3)	33 (5.2)	46 (3.3)
Amputation-free survival at discharge	3185 (90.2)	1334 (90.6)	576 (90.9)	1280 (93.0)

Values in parentheses are percentages unless indicated otherwise; *values are median (i.q.r.). OP, outpatient; Adm, admission; Dis, discharge; Readm, readmission; CLTI, chronic limb-threatening ischaemia.

For patients who followed the outpatient care pathways and were subsequently admitted to an arterial hub hospital for revascularization (3530 and 1473 patients on pathways 4 and 5 respectively), the median times to revascularization were similar, regardless of the type of hospital (hub or spoke) that patients presented to during the initial outpatient visit (median 13 (6–25) days). However, the 634 patients who were admitted to a non-arterial spoke hospital following the initial outpatient visit (pathway 6) tended to experience longer delays to revascularization (median 26 (15–35) days) than patients who followed pathways 4 and 5 (*P* < 0.001). As noted previously, patients who were discharged in the middle of the care pathway (pathway 7) had significantly longer delays to revascularization, with only 14.6 per cent having a procedure within 14 days. The distribution of delays for each pathway are shown in *Fig. S3*.

These figures for each pathway (*Tables 3* and *4*) highlight a distinct difference between arterial hubs and spoke hospitals, regardless of how patients first attended. For the 10 563 patients (64.1 per cent of all patients) whose first admission was to an arterial hub hospital (pathways 1, 4, and 5), just over half underwent revascularization within the times recommended by the Vascular Society of Great Britain and Ireland (VSGBI) (5 days for inpatients, 14 days for outpatients). However, more than three-quarters of patients missed the time targets among the 2417 (14.7 per cent of all patients) whose first admission was to a non-arterial spoke hospital (pathways 2 and 6). The proportion of patients meeting the recommended time targets was statistically significantly lower among patients following pathways 2 and 6, compared with that for patients following pathways 1, 4, and 5 (20.1 *versus* 52.5 per cent; *P* < 0.001).

### Factors associated with time to revascularization

**Fig. 2:**
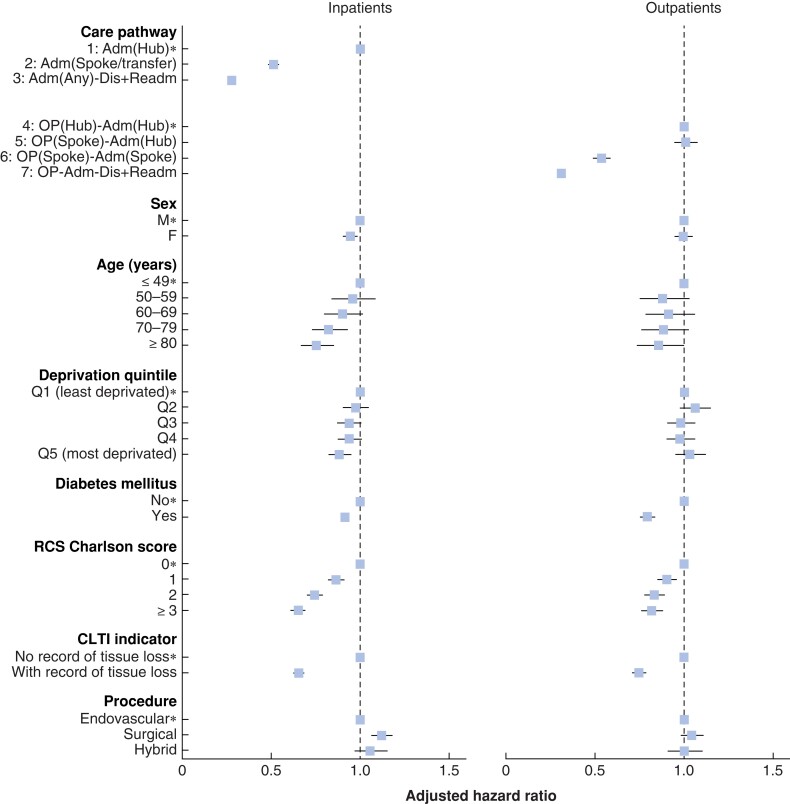
Adjusted hazard ratios for time to revascularization for patient characteristics and care pathway Adjusted hazard ratios, with 95 per cent confidence intervals, estimated using multivariable Cox regression models, with National Health Service Trusts included as random effects. *Reference group. A hazard ratio of less than 1 for a subgroup indicates that the time to revascularization tended to be longer for patients in that subgroup compared with patients in the reference group. Adm, admission; Dis, discharge; Readm, readmission; OP, outpatient; RCS, Royal College of Surgeons; CLTI, chronic limb-threatening ischaemia.

There were some marked differences across patient characteristics in the proportion of patients who exceeded the 5-day target (*Table S4*) and the 14-day target (*Table S5*), notably whether or not a patient presented with tissue loss and an increasing number of co-morbidities. However, patient factors did not fully explain the differences in times to revascularization across the various types of care pathway. *Figure 2* shows the adjusted hazard ratios describing the association between time to revascularization and the various inpatient and outpatient care pathways, as well as the influence of different patient and clinical characteristics. Among patients who followed the inpatient care pathways, longer delays to revascularization were associated with increasing age, patients presenting with tissue loss, and a greater number of co-morbidities (*Fig. 2* and *Table S4*). Among the patients who followed the outpatient care pathways, the associations with longer times were also statistically significant for patients presenting with tissue loss, a greater number of co-morbidities, and a diagnosis of diabetes (*Fig. 2* and *Table S5*).

### Postoperative major amputation and in-hospital death after revascularization

**Fig. 3:**
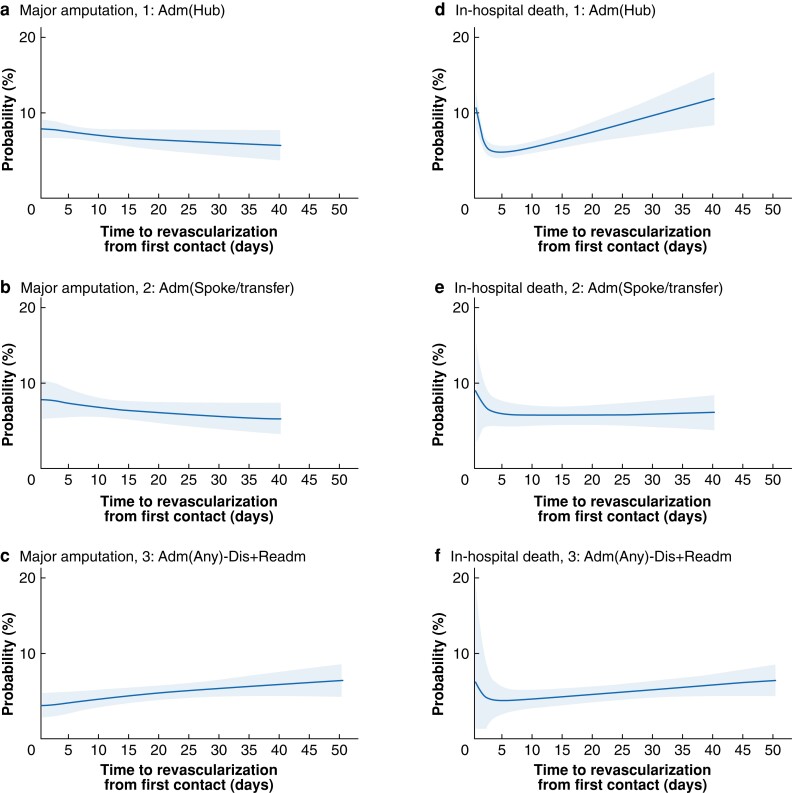
Marginal predicted probability of postoperative major amputation and in-hospital death across the inpatient care pathways against time to revascularization **a–c** Probability of major amputation for pathways 1–3 respectively and **d–f** probability of in-hospital death for pathways 1–3 respectively. Shaded area represents 95 per cent confidence interval. Details of fitted regression models can be found in *Table S6*. Adm, admission; Dis, discharge; Readm, readmission.

**Fig. 4:**
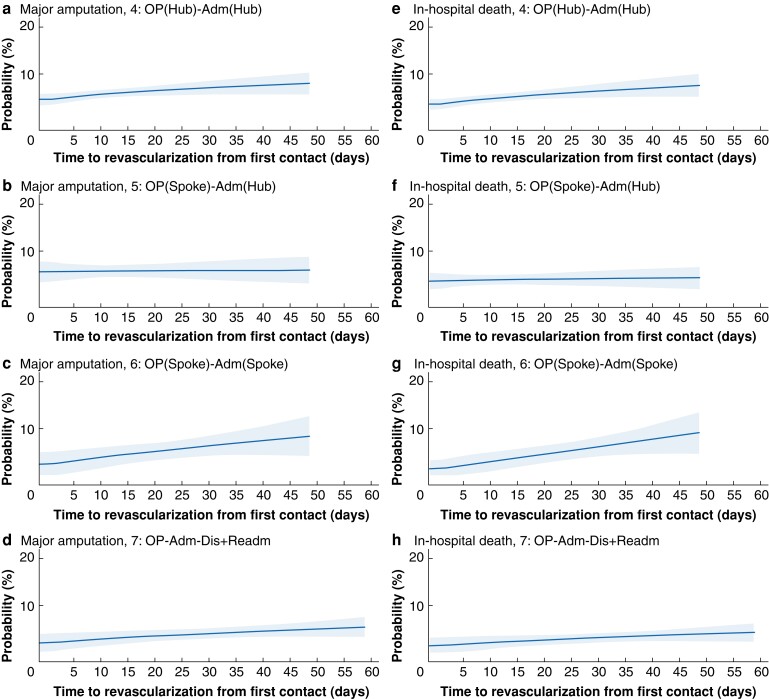
Marginal predicted probability of postoperative major amputation and in-hospital death across the outpatient care pathways against time to revascularization **a–d** Probability of major amputation for pathways 4–7 respectively and **e–h** probability of in-hospital death for pathways 4–7 respectively. Shaded area represents 95 per cent confidence interval. Details of fitted regression models can be found in *Table S6*. OP, outpatient; Adm, admission; Dis, discharge; Readm, readmission.

Overall, 1018 patients (6.2 per cent) underwent major amputation in the postoperative phase within the same admission, whereas 864 (5.2 per cent) died in hospital after revascularization. In all, 14 733 patients (89.4 per cent) were alive and amputation-free at discharge (*Tables 3* and *4*). The univariable relationships between delays to revascularization and the risk of each postoperative outcome after revascularization across care pathways are shown in *Figs S4* and *S5*. Generally, within the time interval containing most patients, longer delays to revascularization were associated with worse postoperative outcomes in most care pathways (*Figs. 3* and *4*), after adjustment for patient and clinical characteristics. The relationship between the adjusted rate of in-hospital amputation and time to revascularization was also qualitatively different for inpatient pathways 1 and 2 compared with the others. As the delay increased, the rate of major amputation decreased slightly for pathways 1 and 2, whereas it increased for outpatient pathways and the inpatient pathway 3.

For in-hospital death, the adjusted relationship with longer time to revascularization was also qualitatively different for the inpatient and outpatient pathways. For patients who were first admitted (pathways 1, 2, and 3), the risk of postoperative death in hospital was lowest when the time to revascularization was between 3 and 7 days, and increased for both shorter and longer delays (*Fig. 3*), the change being greatest for pathway 1. The risk of in-hospital postoperative death for the outpatient pathways was also estimated to increase with longer delays, but these did not have the higher risk associated with the shortest delays (*Fig. 4*). *Table S6* shows the model regression coefficients for these estimates.

### Sensitivity analyses

The results of sensitivity analyses are presented in *Tables S7–S10*. Overall, the results were similar to those for the main analyses. The proportions of patients following outpatient care pathways were changed by varying the 30-day limit to 15 and 60 days, and by changing the outpatient specialties. However, for all scenarios, patients admitted to a non-arterial spoke hospital waited on average about twice as long for revascularization than those admitted to an arterial hub hospital. Results of the Cox and logistic regression models were robust across the sensitivity analyses.

## Discussion

This study used a novel approach to describe the complex care pathways to revascularization for patients with CLTI within the hub-and-spoke models of vascular networks in England. The results highlight a number of issues. It is of concern that patients with CLTI who were first seen at non-arterial spoke hospitals experienced longer delays to accessing revascularization procedures than those who were first seen at an arterial hub hospital. Patients admitted to a non-arterial spoke hospital (pathway 2) waited on average more than twice as long for revascularization than patients admitted to an arterial hub hospital (pathway 1). Similar differences were observed in relation to the outpatient pathways when patients were treated only at a hub or spoke (pathways 4 and 6). There were almost identical times to revascularization among patients who had an initial outpatient assessment, regardless if that was at an arterial hub or a non-arterial spoke, before subsequent admission to an arterial hub unit for revascularization (pathway 4 *versus* 5). Nonetheless, about 45 per cent of patients missed the target of a 14-day maximum delay for patients following outpatient care pathways 4 and 5. Finally, the results suggested that, after around 7 days, longer delays are associated with a slightly but statistically significantly increased risk of postoperative major amputation and in-hospital death. An additional interesting observation was the higher risk of in-hospital death among patients who were admitted as an emergency to an arterial hub (pathway 1) and fairly rapid revascularization was performed within 3 days, which could reflect the likelihood that those treated soonest were the sickest patients, often with the most considerable degree of ischaemia.

The findings regarding time to revascularizsation across care pathways within the hub-and-spoke vascular networks are in agreement with those of previous studies, although few studies have investigated the relationship between delays to revascularization and postoperative outcomes in patients with CLTI. Pankhurst and Edmonds^21^ identified the centralization of UK vascular services as one of the reasons why patients with diabetes and PAD had difficulty accessing specialist vascular services. An organizational survey of UK vascular units reported that some Trusts (32 of 77) had about 1 in 10 patients waiting longer than 48 hours for transfer from a non-arterial spoke unit to an arterial hub unit^14^. The present study supports the survey findings, and highlighted that patients with CLTI who were transferred from a non-arterial spoke hospital to an arterial hub for revascularization (pathways 2 and 6) experienced longer delays to revascularization. An unexpected finding was the longer time to revascularization among patients presenting with tissue loss. It is possible that these patients had more severe co-morbidity or frailty, and so needed longer for investigations and preoperative optimization.

There has been a long-standing concern that late presentation and delayed management of patients with CLTI could contribute to increased major lower limb amputation rates^15^. A Finnish study^22^ reported that a delay of more than 2 weeks from the primary care assessment to revascularization was an independent predictor of major amputation in patients with diabetes and CLTI presenting with tissue loss (odds ratio 3.1, 95 per cent c.i. 1.4 to 6.9), compared with a delay of less than 2 weeks. The UK National Vascular Registry (NVR) 2020 Annual Report^23^ also documented higher in-hospital mortality rates in patients admitted as an emergency whose time from admission to revascularization exceeded 5 days than among those whose preoperative duration of hospital stay was 5 days or less. In the present study, postoperative outcomes were worse when associated with longer delays to revascularization, although patterns varied across care pathways. Among patients who followed the outpatient care pathways, in particular pathways 4 and 6, there were small but positive trends between time to revascularization and the adverse postoperative outcomes of major amputation and in-hospital death. For patients who were initially admitted directly to an arterial hub hospital as an emergency (pathway 1), the risk of in-hospital death was least when revascularization was performed between 3 and 7 days of admission, and then increased markedly as delays lengthened. A possible explanation is that inpatients who experienced delayed revascularization may have had a greater burden of co-morbidity requiring additional time to optimize concurrent medical co-morbidities (cardiac, respiratory, renal, diabetes or infective) before revascularization was attempted. A greater proportion of high-risk patients in this group might also explain the greater risk of death among patients with the shortest times to revascularization. For the inpatients who were discharged and subsequently readmitted for revascularization (pathway 3), longer delays also appeared to be associated with an increased risk of postoperative major amputation after adjusting for other patient characteristics; however, this finding was only marginally statistically significant. Further investigations into the reasons for interim discharge and subsequent readmission for revascularization may be required to improve postoperative outcomes and reduce the amputation rates in this group of patients.

There is always a risk to life or limb in major arterial surgery, and vascular surgery is classified as an urgent care service in the UK^6,10^. Centres of excellence for amputation prevention have been encouraged worldwide for managing patients with CLTI^1^. In 2019, the VSGBI^10,16^ introduced the PAD QIF with a 5-day target from referral to revascularization procedures for patients with CLTI who follow non-elective admission pathways, and a 14-day target for those who follow outpatient pathways. In the present study, the 5-day inpatient target was met in 50.3 per cent of patients who were admitted directly to an arterial hub hospital as an emergency, and in only 18.8 per cent of those who were admitted to a non-arterial spoke hospital preceding transfer to the regional arterial hub centre. Similar patterns were found in respect of the 14-day target among patients who followed the outpatient care pathways. There is room to improve the time to revascularization from specialist healthcare assessment for patients with CLTI in England. The Leicester Vascular Unit instituted a vascular limb salvage clinic on an outpatient basis in 2018, with the aim of meeting the 14-day target, and reported improved 12-month outcomes and reduced amputation rates for patients with CLTI compared with those managed through traditional clinical pathways^24^. Only 42.5 per cent of patients in this study followed outpatient care pathways, which could imply that most patients with CLTI were managed with late presentation, and a further investigation could be of importance.

The main strength of this study is the use of both inpatient and outpatient data for all English NHS hospitals, which enabled the study to capture the complex care pathways in the real world for patients with CLTI in a comprehensive manner. This study included most patients with CLTI in England who required urgent care and underwent their first lower limb revascularization during the study interval within the hub-and-spoke model of vascular networks. This study also has several potential limitations. First, there are no explicit diagnostic codes for CLTI in the version of ICD-10 used by the HES database (in contrast to the modifications used elsewhere^25^). Therefore, a combination of emergency admission, ICD-10 diagnostic codes, and OPCS-4 procedure codes was used to define the study cohort. The study was limited to emergency admissions as the NVR 2021 Annual Report^26^ documented that more than 95 per cent of non-elective lower limb bypass procedures performed in 2019 were due to CLTI (Fontaine score III–IV). This approach omitted patients with CLTI who had an elective revascularization, but the study was considered to capture the majority of patients with CLTI and to be representative of the whole population. Second, the cohort inclusion criteria relied on the 30-day limit and the range of outpatient specialties used to define the outpatient pathways. Sensitivity analyses that replaced the 30-day limit with 15- or 60-day limits showed that the distribution of time to revascularization for the outpatient care pathways was dependent on this limit. However, a 30-day limit was considered a reasonable interval between the outpatient visit and the admission for revascularization. Third, there is a risk of residual confounding owing to unmeasured confounding variables. This might explain the increased risk of in-hospital death for the shortest times to revascularization among patients on care pathway 1. Finally, HES only collects data on secondary care. Delays that occurred in the community between the onset of symptoms and specialist assessment by vascular services were not captured in this study.

## Supplementary Material

znac109_Supplementary_Data
