# Supplementary material for: Delays to revascularization for patients with chronic limb-threatening ischaemia
Source: Br J Surg. 2022 May 11;109(8):717–26. doi: 10.1093/bjs/znac109 (PMC10364726; doi:10.1093/bjs/znac109)

**Supplementary Material**

**Delays to revascularisation for patients with chronic limb-threatening ischaemia**

Q. Li, P. Birmpili, A.S. Johal, S. Waton, A.D. Pherwani, J.R. Boyle, D.A. Cromwell

**Table S1.** ICD-10 codes for chronic limb-threatening ischaemia (CLTI). Note: patients with CLTI were defined using a combination of ICD-10 codes and emergency admission among those undergoing revascularisation procedures.

| **Disease/condition** | **ICD-10 code** | **ICD-10 code description** |
| --- | --- | --- |
| Intermittent claudication | I739 | Peripheral vascular disease, unspecified |
| Severe limb ischaemia | I702 | Atherosclerosis of arteries of extremities  I70.02.1 (with gangrene) |
|  | I771 | Stricture of artery |
|  | I779 | Disorder of arteries and arterioles, unspecified |
|  | L030 | Cellulitis of finger and toe |
|  | L031 | Cellulitis of other parts of limb |
| Diabetes with peripheral circulatory complications | E105 | Type 1 diabetes mellitus with peripheral circulatory complication |
|  | E115 | Type 2 diabetes mellitus with peripheral circulatory complication |
|  | E125 | Malnutrition-related diabetes with peripheral circulatory complication |
|  | E135 | Other specified diabetes mellitus with peripheral circulatory complication |
|  | E145 | Unspecified diabetes mellitus with peripheral circulatory complication |
|  | I792 | Peripheral angiopathy in diseases classified elsewhere (diabetic peripheral angiopathy) |
| Ulceration | L97X | Ulcer of lower limb, not elsewhere classified |
|  | L984 | Chronic ulcer of skin, not elsewhere classified |
| Gangrene | R02X | Gangrene, not elsewhere classified |
| Osteomyelitis | M860 | Acute haematogenous osteomyelitis |
|  | M861 | Other acute osteomyelitis |
|  | M862 | Subacute osteomyelitis |
|  | M863 | Chronic multifocal osteomyelitis |
|  | M864 | Chronic osteomyelitis with draining sinus |
|  | M865 | Other chronic haematogenous osteomyelitis |
|  | M866 | Other chronic osteomyelitis |
|  | M868 | Other osteomyelitis |
|  | M869 | Osteomyelitis, unspecified |
| Emergency admissions in the HES data were identified from the admission method with codes of 21, 22, 23, 24, 28, 2A, 2B, 2D.  Lower limb revascularisations with the following primary diagnoses were considered as non CLTI-related and excluded from the study:   - I71*: Aortic aneurysm and dissection - I723, I724: Other aneurysm - I743, I744, I745: Arterial embolism and thrombosis – acute limb ischaemia | | |

**Table S2.** Office of Population Censuses and Surveys Classification of Surgical Operations and Procedures (OPCS) version 4 codes to define endovascular and surgical lower limb revascularisation, and major amputation

| **Location** | **Code** | **Description** |
| --- | --- | --- |
| **Endovascular revascularisation for lower limb artery** | | |
| Iliac | L541 | Percutaneous transluminal angioplasty of iliac artery |
|  | L544 | Percutaneous transluminal insertion of stent into iliac artery |
| femoral | L631 | Percutaneous transluminal angioplasty of femoral artery |
|  | L635 | Percutaneous transluminal insertion of stent into femoral artery |
| generic | L662 | Percutaneous transluminal stent reconstruction of artery |
|  | L665 | Percutaneous transluminal balloon angioplasty of artery |
|  | L667 | Percutaneous transluminal placement of peripheral stent in artery |
|  | L711 | Percutaneous transluminal angioplasty of artery |
| **Surgical revascularisation for lower limb artery** | | |
| Bypass:  aorta-femoral arteries | L161 | Emergency bypass of aorta by anastomosis of axillary artery to femoral artery |
|  | L162 | Bypass of aorta by anastomosis of axillary artery to femoral artery NEC |
|  | L163 | Bypass of aorta by anastomosis of axillary artery to bilateral femoral arteries |
| Bypass:  Aorta-iliac artery | L206 | Emergency bypass of bifurcation of aorta by anastomosis of aorta to iliac artery NEC |
|  | L216 | Bypass of bifurcation of aorta by anastomosis of aorta to iliac artery NEC |
| Bypass:  Iliac-iliac / femoral artery | L501 | Emergency bypass of common iliac artery by anastomosis of aorta to common iliac artery NEC |
|  | L502 | Emergency bypass of iliac artery by anastomosis of aorta to external iliac artery NEC |
|  | L503 | Emergency bypass of artery of leg by anastomosis of aorta to common femoral artery NEC |
|  | L504 | Emergency bypass of artery of leg by anastomosis of aorta to deep femoral artery NEC |
|  | L505 | Emergency bypass of iliac artery by anastomosis of iliac artery to iliac artery NEC |
|  | L506 | Emergency bypass of artery of leg by anastomosis of iliac artery to femoral artery NEC |
|  | L508 | Other specified other emergency bypass of iliac artery |
|  | L509 | Unspecified other emergency bypass of iliac artery |
|  | L511 | Bypass of common iliac artery by anastomosis of aorta to common iliac artery NEC |
|  | L512 | Bypass of iliac artery by anastomosis of aorta to external iliac artery NEC |
|  | L513 | Bypass of artery of leg by anastomosis of aorta to common femoral artery NEC |
|  | L514 | Bypass of artery of leg by anastomosis of aorta to deep femoral artery NEC |
|  | L515 | Bypass of iliac artery by anastomosis of iliac artery to iliac artery NEC |
|  | L516 | Bypass of artery of leg by anastomosis of iliac artery to femoral artery NEC |
|  | L518 | Other specified other bypass of iliac artery |
|  | L519 | Unspecified other bypass of iliac artery |
| Bypass:  Femoral – femoral / popliteal / tibial / peroneal | L581 | Emergency bypass of femoral artery by anastomosis of femoral artery to femoral artery NEC |
|  | L582 | Emergency bypass of femoral artery by anastomosis of femoral artery to popliteal artery using prosthesis NEC |
|  | L583 | Emergency bypass of femoral artery by anastomosis of femoral artery to popliteal artery using vein graft NEC |
|  | L584 | Emergency bypass of femoral artery by anastomosis of femoral artery to tibial artery using prosthesis NEC |
|  | L585 | Emergency bypass of femoral artery by anastomosis of femoral artery to tibial artery using vein graft NEC |
|  | L586 | Emergency bypass of femoral artery by anastomosis of femoral artery to peroneal artery using prosthesis NEC |
|  | L587 | Emergency bypass of femoral artery by anastomosis of femoral artery to peroneal artery using vein graft NEC |
|  | L588 | Other specified other emergency bypass of femoral artery |
|  | L589 | Unspecified other emergency bypass of femoral artery |
|  | L591 | Bypass of femoral artery by anastomosis of femoral artery to femoral artery NEC |
|  | L592 | Bypass of femoral artery by anastomosis of femoral artery to popliteal artery using prosthesis NEC |
|  | L593 | Bypass of femoral artery by anastomosis of femoral artery to popliteal artery using vein graft NEC |
|  | L594 | Bypass of femoral artery by anastomosis of femoral artery to tibial artery using prosthesis NEC |
|  | L595 | Bypass of femoral artery by anastomosis of femoral artery to tibial artery using vein graft NEC |
|  | L596 | Bypass of femoral artery by anastomosis of femoral artery to peroneal artery using prosthesis NEC |
|  | L597 | Bypass of femoral artery by anastomosis of femoral artery to peroneal artery using vein graft NEC |
|  | L598 | Other specified other bypass of femoral artery |
|  | L599 | Unspecified other bypass of femoral artery |
| Endarterectomy or profundaplasty | L521 | Endarterectomy of iliac artery and patch repair of iliac artery |
|  | L522 | Endarterectomy of iliac artery NEC |
|  | L528 | Other specified reconstruction of iliac artery |
|  | L529 | Unspecified reconstruction of iliac artery |
|  | L601 | Endarterectomy of femoral artery and patch repair of femoral artery |
|  | L602 | Endarterectomy of femoral artery NEC |
|  | L603 | Profundaplasty of femoral artery and patch repair of deep femoral artery |
|  | L604 | Profundaplasty of femoral artery NEC |
|  | L608 | Other specified reconstruction of femoral artery |
|  | L609 | Unspecified reconstruction of femoral artery |
|  | L681 | Endarterectomy and patch repair of artery NEC |
| Major amputation | X09 | Lower limb major amputation |

**Table S3.** Description of the 19 distinct care pathways identified using the Hospital Episode Statistics (HES) outpatient and inpatient data between 2015 and 2019

| Code | Care pathway description | No.^1^ of patients | % |
| --- | --- | --- | --- |
| CP01 | Admitted spoke->revascularised | 782 | 4.7 |
| CP11 | Admitted hub->revascularised | 5 576 | 33.6 |
| CP12 | Admitted hub->discharged^2^->readmitted same hub->revascularised | 1 287 | 7.7 |
| CP13 | Admitted spoke->transferred to a hub->revascularised | 1 010 | 6.1 |
| CP14 | Admitted spoke->transferred to different spoke->revascularised | 13 | 0.1 |
| CP24 | Admitted spoke->discharged->readmitted hub->revascularised | 704 | 4.2 |
| CP25 | Admitted spoke->discharged->readmitted same spoke->revascularised | 129 | 0.8 |
| CP26 | Admitted spoke->discharged->readmitted different spoke->revascularised | 31 | 0.2 |
| CP31 | Admitted spoke->same day transferred to hub->discharged->readmitted to same hub->revascularised | 21 | 0.1 |
| OCP01 | Outpatient**^3^** spoke->Admitted same spoke->revascularised | 323 | 1.9 |
| OCP02 | Outpatient spoke->Admitted different spoke->revascularised | 52 | 0.3 |
| OCP03 | Outpatient spoke->Admitted same spoke->discharged->readmitted same spoke->revascularised | 92 | 0.6 |
| OCP04 | Outpatient spoke->Admitted different spoke->discharged->readmitted same spoke->revascularised | 11 | 0.1 |
| OCP21 | Outpatient hub visit->Admitted same hub->revascularised | 3 533 | 21.3 |
| OCP22 | Outpatient hub visit->Admitted same hub->discharged->readmitted same hub->revascularised | 807 | 4.9 |
| OCP23 | Outpatient spoke->Admitted hub->revascularised | 1 475 | 8.9 |
| OCP24 | Outpatient spoke->Admitted same spoke->transferred to a hub-> revascularised | 267 | 1.6 |
| OCP25 | Outpatient spoke->Admitted hub->discharged->readmitted same hub-> revascularised | 284 | 1.7 |
| OCP26 | Outpatient spoke->Admitted same spoke->discharged->readmitted hub-> revascularised | 222 | 1.3 |
| Total |  | 16 619^3^ |  |

^1^Patients included in the table were not limited to ≤70 days of time to revascularisation.

^2^The maximum gap between the discharge date and the subsequent readmission date was defined to be 30 days.

^3^The maximum gap between the outpatient visits and the subsequent revascularisation related hospital admissions was defined to be 30 days, where the first outpatient visit was the earliest visit within the 30-days window with a specialist in vascular surgery, diabetic medicine, podiatry or general surgery.

**Table S4**. Analyses results of time to revascularisation for patients who followed the inpatient care pathways. Hazard ratios (HR)/adjusted hazard ratios (aHR), 95% confidence Intervals (95% CI) and p values were estimated using univariable and multivariable Cox regression models. The multivariable model comprises covariates listed in the table and the NHS trusts as random effects.

| **Inpatients** | **Patients waiting time>5 days**  **no. (%)** | **Univariable analysis** | | | **Multivariable analysis** | | |
| --- | --- | --- | --- | --- | --- | --- | --- |
|  |  | **HR** | **95% CI** | **p value** | **aHR** | **95% CI** | **p value** |
| Care Pathway |  |  |  | <0.001 |  |  | <0.001 |
| 1: Adm(Hub) | 2 766 (49.7) | 1 |  |  | 1 |  |  |
| 2: Adm(Spoke / transfer) | 1 447 (81.2) | 0.48 | 0.46-0.51 |  | 0.51 | 0.48-0.54 |  |
| 3: Adm(Any)-Dis+Readm | 1 978 (93.0) | 0.30 | 0.29-0.32 |  | 0.28 | 0.26-0.29 |  |
| Sex |  |  |  | 0.025 |  |  | 0.008 |
| Male | 4 021 (65.4) | 1 |  |  | 1 |  |  |
| Female | 2 170 (65.4) | 0.95 | 0.91-0.99 |  | 0.94 | 0.90-0.98 |  |
| Age (years) |  |  |  | <0.001 |  |  | <0.001 |
| <=49 | 185 (60.7) | 1 |  |  | 1 |  |  |
| 50-59 | 614 (58.3) | 1.03 | 0.90-1.17 |  | 0.96 | 0.84-1.09 |  |
| 60-69 | 1 393 (63.4) | 0.94 | 0.84-1.06 |  | 0.90 | 0.80-1.02 |  |
| 70-79 | 1 936 (66.1) | 0.86 | 0.76-0.97 |  | 0.82 | 0.73-0.93 |  |
| 80+ | 2 063 (69.0) | 0.80 | 0.71-0.90 |  | 0.75 | 0.67-0.85 |  |
| Deprivation quintile |  |  |  | 0.427 |  |  | 0.009 |
| Q1 (least deprived) | 842 (63.9) | 1 |  |  | 1 |  |  |
| Q2 | 1 101 (64.8) | 0.99 | 0.92-1.07 |  | 0.97 | 0.90-1.05 |  |
| Q3 | 1 204 (64.9) | 0.99 | 0.93-1.07 |  | 0.94 | 0.87-1.01 |  |
| Q4 | 1 372 (64.6) | 0.99 | 0.93-1.07 |  | 0.94 | 0.87-1.01 |  |
| Q5 (most deprived) | 1 672 (67.6) | 0.95 | 0.89-1.02 |  | 0.88 | 0.82-0.95 |  |
| Diabetes mellitus |  |  |  | <0.001 |  |  | <0.001 |
| No | 2 759 (59.0) | 1 |  |  | 1 |  |  |
| Yes | 3 432 (71.6) | 0.83 | 0.80-0.86 |  | 0.91 | 0.88-0.95 |  |
| RCS Charlson score (diabetes not included) | | | | <0.001 |  |  | <0.001 |
| 0 | 1 785 (57.5) | 1 |  |  | 1 |  |  |
| 1 | 1 841 (63.9) | 0.84 | 0.80-0.88 |  | 0.86 | 0.82-0.91 |  |
| 2 | 1 313 (70.7) | 0.70 | 0.66-0.74 |  | 0.74 | 0.70-0.79 |  |
| 3+ | 1 252 (77.1) | 0.58 | 0.54-0.61 |  | 0.65 | 0.61-0.69 |  |
| CLTI indicator |  |  |  | <0.001 |  |  | <0.001 |
| No record of tissue loss | 2 128 (51.7) | 1 |  |  | 1 |  |  |
| With record of tissue loss | 4 063 (75.9) | 0.65 | 0.63-0.68 |  | 0.66 | 0.63-0.68 |  |
| Procedure |  |  |  | <0.001 |  |  | <0.001 |
| Endovascular | 4 545 (68.4) | 1 |  |  | 1 |  |  |
| Surgical | 1 273 (57.0) | 1.26 | 1.20-1.32 |  | 1.12 | 1.06-1.18 |  |
| Hybrid | 373 (63.1) | 1.14 | 1.05-1.24 |  | 1.06 | 0.97-1.15 |  |

RCS Royal College of Surgeons; CLTI chronic limb-threatening ischaemia.

**Table S5**. Analyses results of time to revascularisation for patients who followed the outpatient care pathways. Hazard ratios (HR)/adjusted hazard ratios (aHR), 95% confidence intervals (95% CI) and p values were estimated using univariable and multivariable Cox regression models. The multivariable model comprises covariates listed in the table and the NHS trusts as random effects.

| **Outpatients** | **Patients waiting time>14 days**  **no. (%)** | **Univariable analysis** | | | **Multivariable analysis** | | |
| --- | --- | --- | --- | --- | --- | --- | --- |
|  |  | **HR** | **95% CI** | **p value** | **aHR** | **95% CI** | **p value** |
| Care Pathway |  |  |  | <0.001 |  |  | <0.001 |
| 4: OP(Hub)-Adm(Hub) | 1 585 (44.9) | 1 |  |  | 1 |  |  |
| 5: OP(Spoke)-Adm(Hub) | 668 (45.3) | 1.05 | 0.99-1.12 |  | 1.01 | 0.95-1.08 |  |
| 6: OP(Spoke)-Adm(Spoke) | 483 (76.2) | 0.51 | 0.47-0.55 |  | 0.54 | 0.49-0.59 |  |
| 7: OP-Adm-Dis+Readm | 1 175 (85.4) | 0.34 | 0.32-0.36 |  | 0.31 | 0.29-0.33 |  |
| Sex |  |  |  | 0.024 |  |  | 0.901 |
| Male | 2 644 (57.2) | 1 |  |  | 1 |  |  |
| Female | 1 267 (52.9) | 1.06 | 1.01-1.11 |  | 1.00 | 0.95-1.05 |  |
| Age (years) |  |  |  | 0.773 |  |  | 0.171 |
| <=49 | 106 (54.4) | 1 |  |  | 1 |  |  |
| 50-59 | 464 (55.4) | 0.95 | 0.81-1.11 |  | 0.88 | 0.75-1.03 |  |
| 60-69 | 932 (57.2) | 0.92 | 0.80-1.07 |  | 0.91 | 0.78-1.06 |  |
| 70-79 | 1 246 (56.2) | 0.92 | 0.79-1.06 |  | 0.88 | 0.76-1.03 |  |
| 80+ | 1 163 (54.5) | 0.93 | 0.80-1.08 |  | 0.86 | 0.74-0.99 |  |
| Deprivation quintile |  |  |  | 0.005 |  |  | 0.161 |
| Q1 (least deprived) | 613 (56.4) | 1 |  |  | 1 |  |  |
| Q2 | 678 (54.7) | 1.10 | 1.02-1.20 |  | 1.06 | 0.98-1.15 |  |
| Q3 | 795 (57.7) | 1.00 | 0.93-1.08 |  | 0.98 | 0.90-1.06 |  |
| Q4 | 841 (57.2) | 0.99 | 0.91-1.07 |  | 0.98 | 0.90-1.06 |  |
| Q5 (most deprived) | 984 (53.5) | 1.08 | 1.01-1.17 |  | 1.03 | 0.95-1.12 |  |
| Diabetes mellitus |  |  |  | <0.001 |  |  | <0.001 |
| No | 1 282 (46.2) | 1 |  |  | 1 |  |  |
| Yes | 2 629 (62.1) | 0.71 | 0.68-0.75 |  | 0.79 | 0.75-0.84 |  |
| RCS Charlson score (diabetes not included) | | |  | <0.001 |  |  | <0.001 |
| 0 | 1 167 (50.4) | 1 |  |  | 1 |  |  |
| 1 | 1 182 (55.1) | 0.88 | 0.83-0.93 |  | 0.90 | 0.85-0.96 |  |
| 2 | 830 (59.7) | 0.79 | 0.75-0.85 |  | 0.83 | 0.78-0.89 |  |
| 3+ | 732 (63.0) | 0.71 | 0.66-0.76 |  | 0.82 | 0.76-0.88 |  |
| CLTI indicator |  |  |  | <0.001 |  |  | <0.001 |
| No record of tissue loss | 1 275 (48.3) | 1 |  |  | 1 |  |  |
| With record of tissue loss | 2 636 (60.3) | 0.76 | 0.73-0.80 |  | 0.75 | 0.71-0.78 |  |
| Procedure |  |  |  | <0.001 |  |  | 0.386 |
| Endovascular | 2 837 (56.7) | 1 |  |  | 1 |  |  |
| Surgical | 823 (53.5) | 1.12 | 1.06-1.19 |  | 1.04 | 0.98-1.11 |  |
| Hybrid | 251 (53.0) | 1.12 | 1.02-1.23 |  | 1.00 | 0.91-1.10 |  |
| RCS Royal College of Surgeons; CLTI chronic limb-threatening ischaemia. | | | | | | | |

**Table S6**. Relationship between postoperative outcomes after revascularisation and patient characteristics among inpatients and outpatients. Adjusted odds ratio (OR), 95% confidence intervals (95% CI) were estimated using mixed effects logistic regression models, with NHS trusts as random effects.

|  |  | **Adjusted OR** | **95% CI** | **P value** |
| --- | --- | --- | --- | --- |
| **Inpatients outcome: major amputation** | | | | |
| Care pathway |  |  |  |  |
| 1: Adm(Hub) |  | 1 | - | - |
| 2: Adm(Spoke / transfer) |  | 0.99 | 0.67-1.46 | 0.959 |
| 3: Adm(Any)-Dis+Readm |  | 0.37 | 0.22-0.65 | <0.001 |
| Interaction between care pathway and time to revascularisation | | | | |
| 1: Adm(Hub) | Ln(time) ^2^ | 0.97 | 0.94-1.01 | 0.156 |
| 2: Adm(Spoke / transfer) | Ln(time) ^2^ | 0.97 | 0.92-1.02 | 0.231 |
| 3: Adm(Any)-Dis+Readm | Ln(time) ^2^ | 1.05 | 1.00-1.11 | 0.058 |
|  |  |  |  |  |
| **Inpatients outcome: in-hospital death** | | | | |
| Care pathway |  |  |  |  |
| 1: Adm(Hub) |  | 1 | - | - |
| 2: Adm(Spoke / transfer) |  | 1.09 | 0.61-1.96 | 0.765 |
| 3: Adm(Any)-Dis+Readm |  | 0.67 | 0.13-3.42 | 0.634 |
| Interaction between care pathway and time to revascularisation | | | | |
| 1: Adm(Hub) | Ln(time) | 0.57 | 0.46-0.69 | <0.001 |
|  | Ln(time) ^2^ | 1.22 | 1.14-1.31 | <0.001 |
| 2: Adm(Spoke / transfer) | Ln(time) | 0.78 | 0.47-1.30 | 0.344 |
|  | Ln(time) ^2^ | 1.05 | 0.92-1.20 | 0.447 |
| 3: Adm(Any)-Dis+Readm | Ln(time) | 0.73 | 0.22-2.42 | 0.601 |
|  | Ln(time) ^2^ | 1.11 | 0.89-1.39 | 0.365 |
|  |  |  |  |  |
| **Outpatients outcome: major amputation** | | | | |
| Care pathway |  |  |  |  |
| 4: OP(Hub)-Adm(Hub) |  | 1 | - | - |
| 5: OP(Spoke)-Adm(Hub) |  | 1.25 | 0.75-2.08 | 0.386 |
| 6: OP(Spoke)-Adm(Spoke) |  | 0.51 | 0.16-1.64 | 0.258 |
| 7: OP-Adm-Dis+Readm |  | 0.45 | 0.17-1.21 | 0.115 |
| Interaction between care pathway and time to revascularisation | | | | |
| 4: OP(Hub)-Adm(Hub) | Ln(time) ^2^ | 1.04 | 1.01-1.08 | 0.024 |
| 5: OP(Spoke)-Adm(Hub) | Ln(time) ^2^ | 1.00 | 0.95-1.06 | 0.863 |
| 6: OP(Spoke)-Adm(Spoke) | Ln(time) ^2^ | 1.09 | 0.99-1.21 | 0.084 |
| 7: OP-Adm-Dis+Readm | Ln(time) ^2^ | 1.06 | 0.98-1.14 | 0.121 |
|  |  |  |  |  |
| **Outpatients outcome: in-hospital death** | | | | |
| Care pathway |  |  |  |  |
| 4: OP(Hub)-Adm(Hub) |  | 1 | - | - |
| 5: OP(Spoke)-Adm(Hub) |  | 1.04 | 0.57-1.89 | 0.91 |
| 6: OP(Spoke)-Adm(Spoke) |  | 0.39 | 0.10-1.54 | 0.177 |
| 7: OP-Adm-Dis+Readm |  | 0.42 | 0.13-1.37 | 0.151 |
| Interaction between care pathway and time to revascularisation | | | | |
| 4: OP(Hub)-Adm(Hub) | Ln(time) ^2^ | 1.06 | 1.02-1.10 | 0.006 |
| 5: OP(Spoke)-Adm(Hub) | Ln(time) ^2^ | 1.01 | 0.95-1.08 | 0.692 |
| 6: OP(Spoke)-Adm(Spoke) | Ln(time) ^2^ | 1.14 | 1.02-1.28 | 0.022 |
| 7: OP-Adm-Dis+Readm | Ln(time) ^2^ | 1.07 | 0.98-1.17 | 0.148 |

**Table S7.** Summary on frequency, time to revascularisation, postoperative outcomes of major amputation and in-hospital death in four scenarios of sensitivity analyses, by care pathway. The sensitivity analyses are:

1: additionally included patients with a primary diagnostic code for acute limb ischaemia and secondary diagnostic codes for CLTI; the interval between outpatient visit and admission was limit to 30 days

2: the interval between outpatient visit and admission was limited to 15 days

3: the interval between outpatient visit and admission was limited to 60 days; patients with defined time to revascularisation>100 days were excluded

4: limit specialist review to vascular surgery only; the interval between outpatient visit and admission was limit to 30 days

|  | **Sensitivity analysis 1** | **Sensitivity analysis 2** | | **Sensitivity analysis 3** | | **Sensitivity analysis 4** | |  |
| --- | --- | --- | --- | --- | --- | --- | --- | --- |
| **No. of patients (%)** | | |  | |  | |  | |
| Inpatients | 11 235 (58.3) | 10 464 (63.3) | | 8 556 (51.7) | | 12 045 (72.9) | |  |
| Outpatients | 8 043 (41.7) | 6 059 (36.7) | | 7 989 (48.3) | | 4 471 (27.1) | |  |
| 1: Adm(Hub) | 6 745 (35.0) | 6 154 (37.3) | | 5 002 (30.2) | | 7 193 (43.6) | |  |
| 2: Adm(Spoke / transfer) | 2 064 (10.7) | 1 889 (11.4) | | 1 668 (10.1) | | 2 158 (13.1) | |  |
| 3: Adm(Any)-Dis+Readm | 2 426 (12.6) | 2 421 (14.7) | | 1 886 (11.4) | | 2 694 (16.3) | |  |
| 4: OP(Hub)-Adm(Hub) | 4 019 (20.9) | 3 123 (18.9) | | 3 912 (23.6) | | 2 360 (14.3) | |  |
| 5: OP(Spoke)-Adm(Hub) | 1 731 ( 9.0) | 1 299 ( 7.9) | | 1 650 (10.0) | | 1 026 ( 6.2) | |  |
| 6: OP(Spoke)-Adm(Spoke) | 725 ( 3.8) | 534 ( 3.2) | | 766 ( 4.6) | | 257 ( 1.6) | |  |
| 7: OP-Adm-Dis+Readm | 1 568 ( 8.1) | 1 103 ( 6.7) | | 1 661 (10.0) | | 828 ( 5.0) | |  |
| **Time to revascularisation, median (IQR) days** | | | | |  | |  | |
| 1: Adm(Hub) | 5 ( 2- 9) | 5 ( 2-10) | | 5 ( 2-10) | | 6 ( 2-10) | |  |
| 2: Adm(Spoke / transfer) | 12 ( 6-19) | 12 ( 7-19) | | 12 ( 7-20) | | 12 ( 7-19) | |  |
| 3: Adm(Any)-Dis+Readm | 20 (12-30) | 20 (12-30) | | 21 (12-31) | | 20 (12-30) | |  |
| 4: OP(Hub)-Adm(Hub) | 13 ( 6-25) | 8 ( 4-14) | | 22 ( 7-46) | | 9 ( 4-21) | |  |
| 5: OP(Spoke)-Adm(Hub) | 13 ( 6-24) | 8 ( 5-14) | | 22 ( 7-46) | | 10 ( 5-22) | |  |
| 6: OP(Spoke)-Adm(Spoke) | 25 (15-35) | 17 (10-24) | | 44 (24-60) | | 22 (13-32) | |  |
| 7: OP-Adm-Dis+Readm | 32 (21-43) | 24 (15-35) | | 46 (28-64) | | 29 (18-41) | |  |
| **Major amputation, no. (%)** | | |  | |  | |  | |
| 1: Adm(Hub) | 478 (7.1) | 438 (7.1) | | 369 (7.4) | | 501 (7.0) | |  |
| 2: Adm(Spoke / transfer) | 142 (6.9) | 130 (6.9) | | 113 (6.8) | | 152 (7.0) | |  |
| 3: Adm(Any)-Dis+Readm | 125 (5.2) | 117 (4.8) | | 97 (5.1) | | 142 (5.3) | |  |
| 4: OP(Hub)-Adm(Hub) | 229 (5.7) | 176 (5.6) | | 220 (5.6) | | 133 (5.6) | |  |
| 5: OP(Spoke)-Adm(Hub) | 109 (6.3) | 76 (5.9) | | 102 (6.2) | | 57 (5.6) | |  |
| 6: OP(Spoke)-Adm(Spoke) | 39 (5.4) | 30 (5.6) | | 47 (6.1) | | 8 (3.1) | |  |
| 7: OP-Adm-Dis+Readm | 66 (4.2) | 53 (4.8) | | 74 (4.5) | | 29 (3.5) | |  |
| **In hospital death, no. (%)** | | |  | |  | |  | |
| 1: Adm(Hub) | 401 (6.0) | 368 (6.0) | | 307 (6.1) | | 401 (5.6) | |  |
| 2: Adm(Spoke / transfer) | 136 (6.6) | 121 (6.4) | | 106 (6.4) | | 140 (6.5) | |  |
| 3: Adm(Any)-Dis+Readm | 123 (5.1) | 122 (5.0) | | 104 (5.5) | | 132 (4.9) | |  |
| 4: OP(Hub)-Adm(Hub) | 185 (4.6) | 141 (4.5) | | 187 (4.8) | | 114 (4.8) | |  |
| 5: OP(Spoke)-Adm(Hub) | 77 (4.5) | 55 (4.2) | | 70 (4.2) | | 50 (4.9) | |  |
| 6: OP(Spoke)-Adm(Spoke) | 36 (5.0) | 25 (4.7) | | 41 (5.4) | | 6 (2.3) | |  |
| 7: OP-Adm-Dis+Readm | 55 (3.5) | 35 (3.2) | | 57 (3.4) | | 25 (3.0) | |  |

**Table S8.** Analyses results of time to revascularisation for patients who followed inpatient pathways in four scenarios for sensitivity analyses. Adjusted hazard ratio and 95% confidence interval (95% CI) were estimated using multivariable Cox regression models including covariates listed in the table and the NHS trusts as random effects. The cohort settings were the same with that in the main context, except the one(s) specified in each scenario*.* See Supplementary Table 7 for definitions of sensitivity analysis.

| **Inpatients** | **Adjusted Hazard Ratio (95% CI)** | | | |
| --- | --- | --- | --- | --- |
|  | **Sensitivity analysis** 1 | **Sensitivity analysis** 2 | **Sensitivity analysis** 3 | **Sensitivity analysis** 4 |
| Care Pathway |  |  |  |  |
| 1: Adm(Hub) | 1 | 1 | 1 | 1 |
| 2: Adm(Spoke / transfer) | 0.49 (0.47-0.52) | 0.51 (0.48-0.54) | 0.50 (0.47-0.53) | 0.51 (0.49-0.54) |
| 3: Adm(Any)-Dis+Readm | 0.27 (0.25-0.28) | 0.28 (0.26-0.29) | 0.28 (0.27-0.3) | 0.27 (0.26-0.28) |
| Sex |  |  |  |  |
| Male | 1 | 1 | 1 | 1 |
| Female | 0.94 (0.9-0.98) | 0.94 (0.91-0.98) | 0.96 (0.92-1) | 0.94 (0.9-0.98) |
| Age (years) |  |  |  |  |
| <=49 | 1 | 1 | 1 | 1 |
| 50-59 | 0.96 (0.85-1.07) | 0.94 (0.83-1.06) | 0.86 (0.75-0.99) | 0.92 (0.82-1.04) |
| 60-69 | 0.89 (0.80-0.99) | 0.90 (0.80-1.01) | 0.85 (0.75-0.97) | 0.88 (0.79-0.98) |
| 70-79 | 0.82 (0.73-0.91) | 0.83 (0.74-0.93) | 0.8 (0.70-0.91) | 0.81 (0.73-0.90) |
| 80+ | 0.75 (0.68-0.84) | 0.75 (0.67-0.84) | 0.73 (0.64-0.83) | 0.75 (0.67-0.84) |
| Deprivation quintile |  |  |  |  |
| Q1 (least deprived) | 1 | 1 | 1 | 1 |
| Q2 | 0.96 (0.90-1.03) | 0.98 (0.92-1.06) | 0.98 (0.90-1.05) | 0.99 (0.92-1.05) |
| Q3 | 0.93 (0.87-0.99) | 0.93 (0.87-1.00) | 0.95 (0.88-1.02) | 0.96 (0.90-1.02) |
| Q4 | 0.92 (0.86-0.99) | 0.93 (0.87-1.00) | 0.95 (0.88-1.02) | 0.95 (0.89-1.01) |
| Q5 (most deprived) | 0.88 (0.82-0.94) | 0.89 (0.83-0.96) | 0.88 (0.81-0.94) | 0.90 (0.85-0.96) |
| Diabetes mellitus |  |  |  |  |
| No | 1 | 1 | 1 | 1 |
| Yes | 0.91 (0.87-0.95) | 0.91 (0.88-0.95) | 0.93 (0.89-0.98) | 0.93 (0.9-0.97) |
| RCS Charlson score (diabetes not included) | |  |  |  |
| 0 | 1 | 1 | 1 | 1 |
| 1 | 0.87 (0.83-0.92) | 0.86 (0.82-0.91) | 0.83 (0.78-0.87) | 0.87 (0.83-0.91) |
| 2 | 0.76 (0.72-0.80) | 0.74 (0.70-0.78) | 0.74 (0.70-0.79) | 0.75 (0.71-0.79) |
| 3+ | 0.67 (0.63-0.71) | 0.65 (0.62-0.69) | 0.63 (0.59-0.67) | 0.68 (0.64-0.72) |
| CLTI indicator |  |  |  |  |
| No record of tissue loss | 1 | 1 | 1 | 1 |
| With record of tissue loss | 0.64 (0.61-0.66) | 0.66 (0.63-0.68) | 0.66 (0.63-0.69) | 0.66 (0.64-0.69) |
| Procedure |  |  |  |  |
| Endovascular | 1 | 1 | 1 | 1 |
| Surgical | 1.16 (1.10-1.21) | 1.13 (1.07-1.18) | 1.11 (1.05-1.17) | 1.11 (1.06-1.17) |
| Hybrid | 1.07 (0.99-1.15) | 1.03 (0.95-1.12) | 1.05 (0.96-1.15) | 1.06 (0.98-1.15) |

RCS Royal College of Surgeons; CLTI chronic limb-threatening ischaemia.

**Table S9.** Analyses results of time to revascularisation for patients who followed outpatient pathways in four scenarios for sensitivity analyses. Adjusted hazard ratios and 95% confidence intervals (95% CI) were estimated using multilevel Cox regression models including covariates listed in the table and the NHS trusts as random effects. The cohort settings were the same with that in the main context, except the one(s) specified in each scenario. See Supplementary Table 7 for definitions of sensitivity analysis.

| **Outpatients** | **Adjusted Hazard Ratio (95% CI)** | | | |
| --- | --- | --- | --- | --- |
|  | **Sensitivity analysis 1** | **Sensitivity analysis 2** | **Sensitivity analysis 3** | **Sensitivity analysis 4** |
| Care Pathway |  |  |  |  |
| 4: OP(Hub)-Adm(Hub) | 1 | 1 | 1 | 1 |
| 5: OP(Spoke)-Adm(Hub) | 1.00 (0.94-1.06) | 1.02 (0.95-1.10) | 1.01 (0.95-1.07) | 0.96 (0.89-1.04) |
| 6: OP(Spoke)-Adm(Spoke) | 0.53 (0.48-0.57) | 0.46 (0.42-0.51) | 0.60 (0.55-0.65) | 0.50 (0.44-0.57) |
| 7: OP-Adm-Dis+Readm | 0.31 (0.29-0.33) | 0.26 (0.24-0.28) | 0.44 (0.41-0.47) | 0.31 (0.28-0.34) |
| Sex |  |  |  |  |
| Male | 1 | 1 | 1 | 1 |
| Female | 1.00 (0.95-1.04) | 0.96 (0.91-1.02) | 1.01 (0.96-1.06) | 0.99 (0.93-1.05) |
| Age (years) |  |  |  |  |
| <=49 | 1 | 1 | 1 | 1 |
| 50-59 | 0.92 (0.79-1.06) | 0.78 (0.66-0.93) | 1.02 (0.88-1.18) | 0.81 (0.66-0.99) |
| 60-69 | 0.93 (0.81-1.07) | 0.86 (0.73-1.01) | 1.06 (0.93-1.23) | 0.84 (0.69-1.01) |
| 70-79 | 0.90 (0.78-1.03) | 0.79 (0.67-0.93) | 1.00 (0.87-1.15) | 0.79 (0.66-0.96) |
| 80+ | 0.88 (0.76-1.01) | 0.77 (0.66-0.91) | 1.01 (0.88-1.16) | 0.74 (0.61-0.9) |
| Deprivation quintile |  |  |  |  |
| Q1 (least deprived) | 1 | 1 | 1 | 1 |
| Q2 | 1.04 (0.96-1.12) | 1.04 (0.95-1.13) | 1.01 (0.93-1.09) | 1.05 (0.94-1.16) |
| Q3 | 0.97 (0.90-1.04) | 0.97 (0.89-1.06) | 0.97 (0.9-1.05) | 0.96 (0.87-1.07) |
| Q4 | 0.96 (0.89-1.04) | 0.97 (0.89-1.06) | 1.02 (0.95-1.11) | 0.98 (0.88-1.09) |
| Q5 (most deprived) | 1.03 (0.95-1.11) | 0.99 (0.91-1.08) | 1.02 (0.94-1.1) | 1.05 (0.95-1.16) |
| Diabetes mellitus |  |  |  |  |
| No | 1 | 1 | 1 | 1 |
| Yes | 0.78 (0.75-0.82) | 0.77 (0.72-0.81) | 0.81 (0.77-0.86) | 0.91 (0.85-0.97) |
| RCS Charlson score (diabetes not included) | |  |  |  |
| 0 | 1 | 1 | 1 | 1 |
| 1 | 0.91 (0.86-0.96) | 0.85 (0.80-0.91) | 0.89 (0.84-0.94) | 0.91 (0.84-0.98) |
| 2 | 0.83 (0.78-0.89) | 0.78 (0.72-0.84) | 0.83 (0.78-0.89) | 0.87 (0.8-0.95) |
| 3+ | 0.81 (0.76-0.87) | 0.77 (0.71-0.84) | 0.78 (0.73-0.84) | 0.82 (0.74-0.9) |
| CLTI indicator |  |  |  |  |
| No record of tissue loss | 1 | 1 | 1 | 1 |
| With record of tissue loss | 0.74 (0.7-0.78) | 0.75 (0.71-0.79) | 0.76 (0.73-0.8) | 0.79 (0.74-0.84) |
| Procedure |  |  |  |  |
| Endovascular | 1 | 1 | 1 | 1 |
| Surgical | 1.03 (0.98-1.09) | 0.96 (0.90-1.02) | 1.01 (0.95-1.06) | 1.00 (0.94-1.08) |
| Hybrid | 1.02 (0.93-1.11) | 0.96 (0.86-1.06) | 1.02 (0.93-1.12) | 0.93 (0.83-1.05) |

RCS Royal College of Surgeons; CLTI chronic limb-threatening ischaemia.

**Table S10.** Analyses results of postoperative outcomes after revascularisation among inpatients and outpatients in four scenarios for sensitivity analyses. Adjusted Odds Ratio and 95% confidence interval (95% CI) of care pathway and their interaction with time to revascularisation were estimated using mixed effects logistic regression models. See Supplementary Table 7 for definitions of sensitivity analysis.

|  |  | **Adjusted Odds Ratio (95% CI)** | | | | | | | |
| --- | --- | --- | --- | --- | --- | --- | --- | --- | --- |
|  |  | **Sensitivity analysis 1** | | **Sensitivity analysis 2** | | **Sensitivity analysis 3** | | **Sensitivity analysis 4** | |
| **Inpatients outcome: major amputation** | | |  | |  | |  | |  |
| Care pathway |  |  | |  | |  | |  | |
| 1: Adm(Hub) |  | 1 | | 1 | | 1 | | 1 | |
| 2: Adm(Spoke / transfer) |  | 0.94 (0.65-1.34) | | 1.03 (0.70-1.50) | | 0.98 (0.65-1.46) | | 1.02 (0.72-1.45) | |
| 3: Adm(Any)-Dis+Readm |  | 0.40 (0.24-0.66) | | 0.37 (0.22-0.63) | | 0.45 (0.26-0.78) | | 0.42 (0.26-0.67) | |
| Interaction between care pathway and time to revascularisation | | | | | | | | |  |
| 1: Adm(Hub) | Ln(time) ^2^ | 0.98 (0.95-1.01) | | 0.99 (0.95-1.02) | | 0.97 (0.94-1.01) | | 0.98 (0.95-1.02) | |
| 2: Adm(Spoke / transfer) | Ln(time) ^2^ | 0.98 (0.94-1.02) | | 0.97 (0.93-1.02) | | 0.96 (0.92-1.01) | | 0.99 (0.94-1.03) | |
| 3: Adm(Any)-Dis+Readm | Ln(time) ^2^ | 1.05 (1.00-1.10) | | 1.05 (1.00-1.11) | | 1.03 (0.98-1.08) | | 1.05 (1.01-1.10) | |
|  |  |  | |  | |  | |  | |
| **Inpatients outcome: in-hospital death** | | |  | |  | |  | |  |
| Care pathway |  |  | |  | |  | |  | |
| 1: Adm(Hub) |  | 1 | | 1 | | 1 | | 1 | |
| 2: Adm(Spoke / transfer) |  | 1.18 (0.71-1.95) | | 1.03 (0.58-1.84) | | 1.00 (0.53-1.88) | | 1.12 (0.64-1.93) | |
| 3: Adm(Any)-Dis+Readm |  | 0.73 (0.16-3.32) | | 0.49 (0.09-2.65) | | 0.53 (0.09-3.07) | | 0.76 (0.17-3.47) | |
| Interaction between care pathway and time to revascularisation | | | | |  | |  | |  |
| 1: Adm(Hub) | Ln(time) | 0.58 (0.48-0.69) | | 0.59 (0.49-0.72) | | 0.56 (0.46-0.69) | | 0.60 (0.50-0.73) | |
|  | Ln(time) ^2^ | 1.21 (1.14-1.29) | | 1.21 (1.13-1.29) | | 1.22 (1.14-1.30) | | 1.21 (1.14-1.29) | |
| 2: Adm(Spoke / transfer) | Ln(time) | 0.81 (0.51-1.27) | | 0.82 (0.50-1.36) | | 0.84 (0.49-1.45) | | 0.86 (0.54-1.39) | |
|  | Ln(time) ^2^ | 1.04 (0.92-1.17) | | 1.05 (0.93-1.19) | | 1.03 (0.90-1.18) | | 1.04 (0.93-1.18) | |
| 3: Adm(Any)-Dis+Readm | Ln(time) | 0.63 (0.21-1.95) | | 0.88 (0.26-3.03) | | 0.73 (0.21-2.49) | | 0.67 (0.22-2.05) | |
|  | Ln(time) ^2^ | 1.15 (0.93-1.41) | | 1.08 (0.86-1.35) | | 1.13 (0.91-1.40) | | 1.14 (0.92-1.40) | |
|  |  |  | |  | |  | |  | |
| **Outpatients outcome: major amputation** | | |  | |  | |  | |  |
| Care pathway |  |  | |  | |  | |  | |
| 4: OP(Hub)-Adm(Hub) |  | 1 | | 1 | | 1 | | 1 | |
| 5: OP(Spoke)-Adm(Hub) |  | 1.26 (0.80-2.00) | | 1.38 (0.82-2.32) | | 1.15 (0.71-1.86) | | 1.09 (0.60-1.97) | |
| 6: OP(Spoke)-Adm(Spoke) |  | 0.52 (0.18-1.53) | | 0.82 (0.29-2.36) | | 0.45 (0.15-1.39) | | 0.70 (0.11-4.47) | |
| 7: OP-Adm-Dis+Readm |  | 0.54 (0.22-1.33) | | 0.47 (0.18-1.22) | | 0.48 (0.19-1.17) | | 0.16 (0.04-0.71) | |
| Interaction between care pathway and time to revascularisation | | | | | | | | |  |
| 4: OP(Hub)-Adm(Hub) | Ln(time) ^2^ | 1.03 (1.00-1.07) | | 1.07 (1.02-1.12) | | 1.02 (0.99-1.04) | | 1.04 (0.99-1.09) | |
| 5: OP(Spoke)-Adm(Hub) | Ln(time) ^2^ | 1.01 (0.97-1.06) | | 1.00 (0.92-1.08) | | 1.01 (0.97-1.04) | | 1.01 (0.95-1.08) | |
| 6: OP(Spoke)-Adm(Spoke) | Ln(time) ^2^ | 1.08 (0.99-1.19) | | 1.07 (0.96-1.19) | | 1.08 (1.00-1.16) | | 0.99 (0.82-1.19) | |
| 7: OP-Adm-Dis+Readm | Ln(time) ^2^ | 1.04 (0.97-1.11) | | 1.08 (1.00-1.18) | | 1.04 (0.98-1.10) | | 1.13 (1.01-1.26) | |
|  |  |  | |  | |  | |  | |
| **Outpatients outcome: in-hospital death** | |  | |  | |  | |  | |
| Care pathway |  |  | |  | |  | |  | |
| 4: OP(Hub)-Adm(Hub) |  | 1 | | 1 | | 1 | | 1 | |
| 5: OP(Spoke)-Adm(Hub) |  | 1.32 (0.78-2.27) | | 1.13 (0.60-2.10) | | 0.98 (0.55-1.73) | | 0.87 (0.44-1.72) | |
| 6: OP(Spoke)-Adm(Spoke) |  | 0.36 (0.10-1.31) | | 0.59 (0.17-2.06) | | 0.21 (0.05-0.93) | | 0.06 (0.01-1.84) | |
| 7: OP-Adm-Dis+Readm |  | 0.49 (0.17-1.41) | | 0.46 (0.14-1.52) | | 0.38 (0.13-1.12) | | 0.42 (0.10-1.72) | |
| Interaction between care pathway and time to revascularisation | | | | | | | | |  |
| 4: OP(Hub)-Adm(Hub) | Ln(time) ^2^ | 1.06 (1.02-1.10) | | 1.07 (1.01-1.13) | | 1.03 (1.00-1.06) | | 1.04 (0.99-1.09) | |
| 5: OP(Spoke)-Adm(Hub) | Ln(time) ^2^ | 0.98 (0.93-1.04) | | 0.99 (0.90-1.08) | | 1.00 (0.95-1.04) | | 1.03 (0.96-1.11) | |
| 6: OP(Spoke)-Adm(Spoke) | Ln(time) ^2^ | 1.14 (1.03-1.27) | | 1.11 (0.98-1.26) | | 1.14 (1.04-1.25) | | 1.22 (0.93-1.61) | |
| 7: OP-Adm-Dis+Readm | Ln(time) ^2^ | 1.06 (0.98-1.15) | | 1.07 (0.96-1.18) | | 1.05 (0.99-1.13) | | 1.04 (0.93-1.16) | |

**Figure S1**. Stacked chart of care pathways between April 2015 and March 2019, by residence region in England

**Figure S2.** Boxplots of median (IQR) time to revascularisation from the point of first contact for the 19 pathways. Note data beyond the upper whiskers (outside values) are not presented; the red line indicates time at 5 days and orange line at 14 days. See Supplementary Table 3 for description of each care pathway code (e.g., CP01)


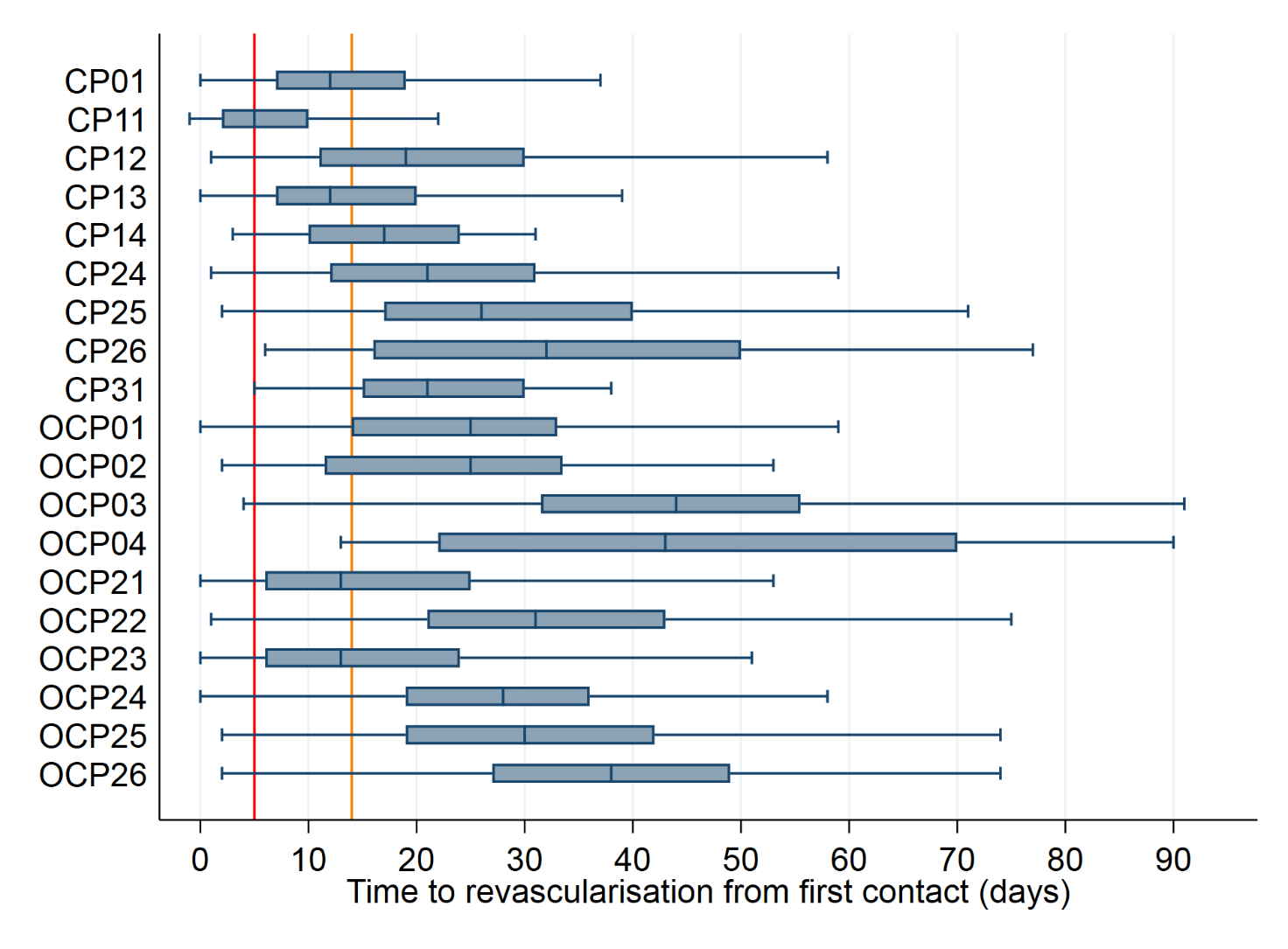


**Figure S3.** Kaplan-Meier estimator of probability of revascularisation over time (days) from the first contact with vascular services, by care pathway


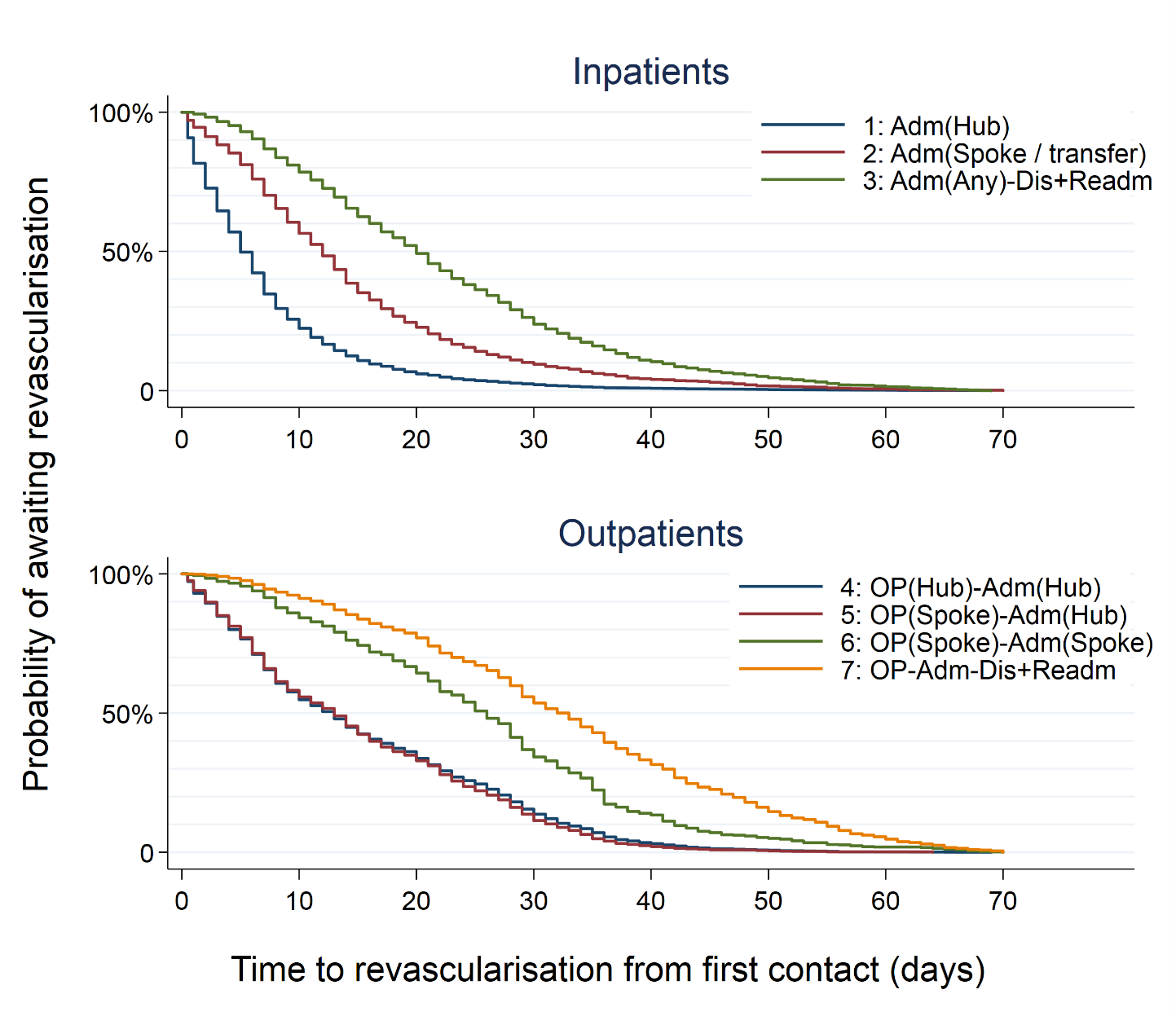


**Figure S4**. Estimated rates of postoperative major amputation (blue line) and in-hospital death (red line) across the inpatient care pathways against time to revascularisation, shaded with 95% confidence interval. Rates were estimated using a nearest neighbour smoother (RUNNING).


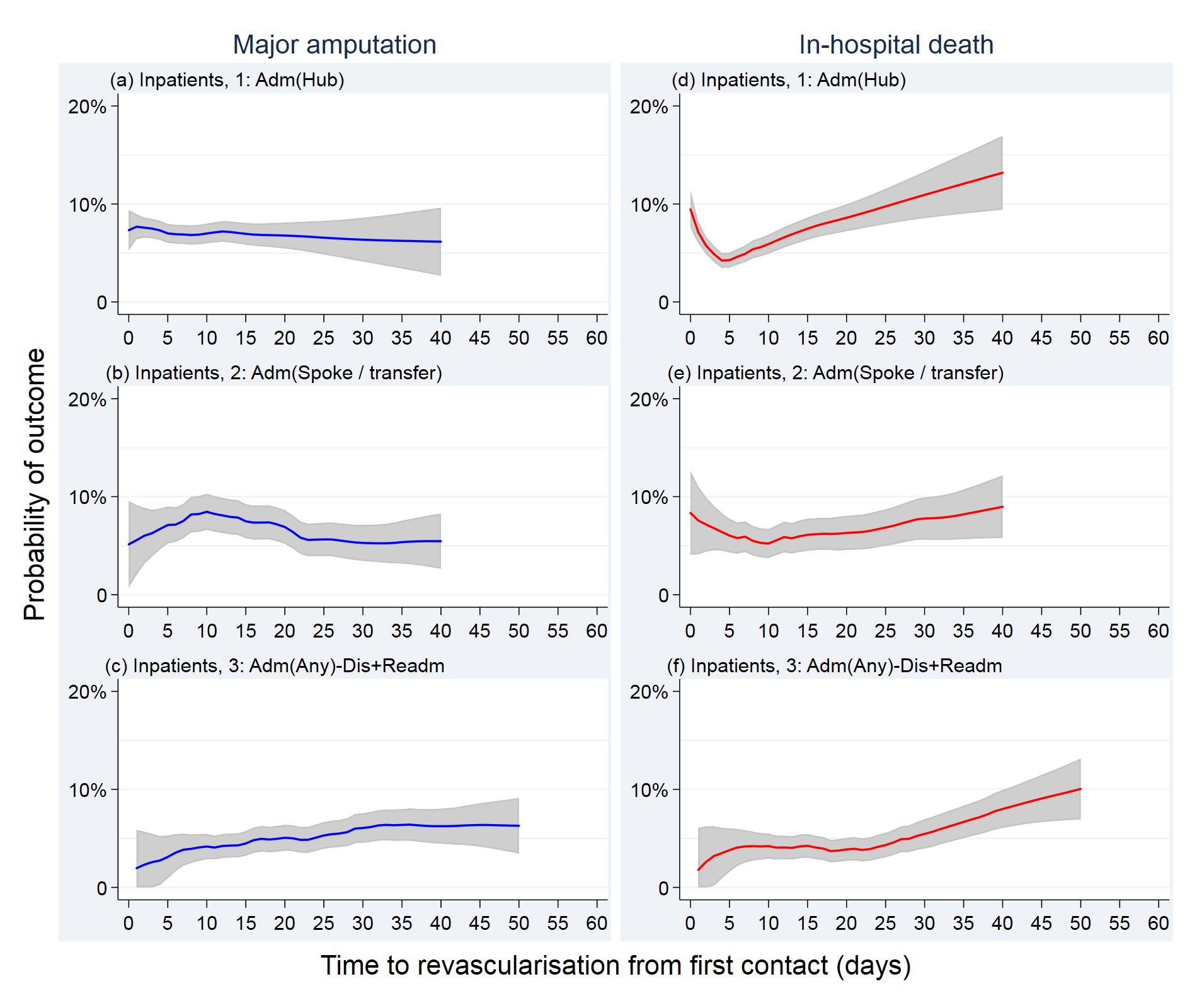


**Figure S5**. Estimated rates of postoperative major amputation (blue line) and in-hospital death (red line) across the outpatient care pathways against time to revascularisation, shaded with 95% confidence interval. Rates were estimated using a nearest neighbour smoother (RUNNING).


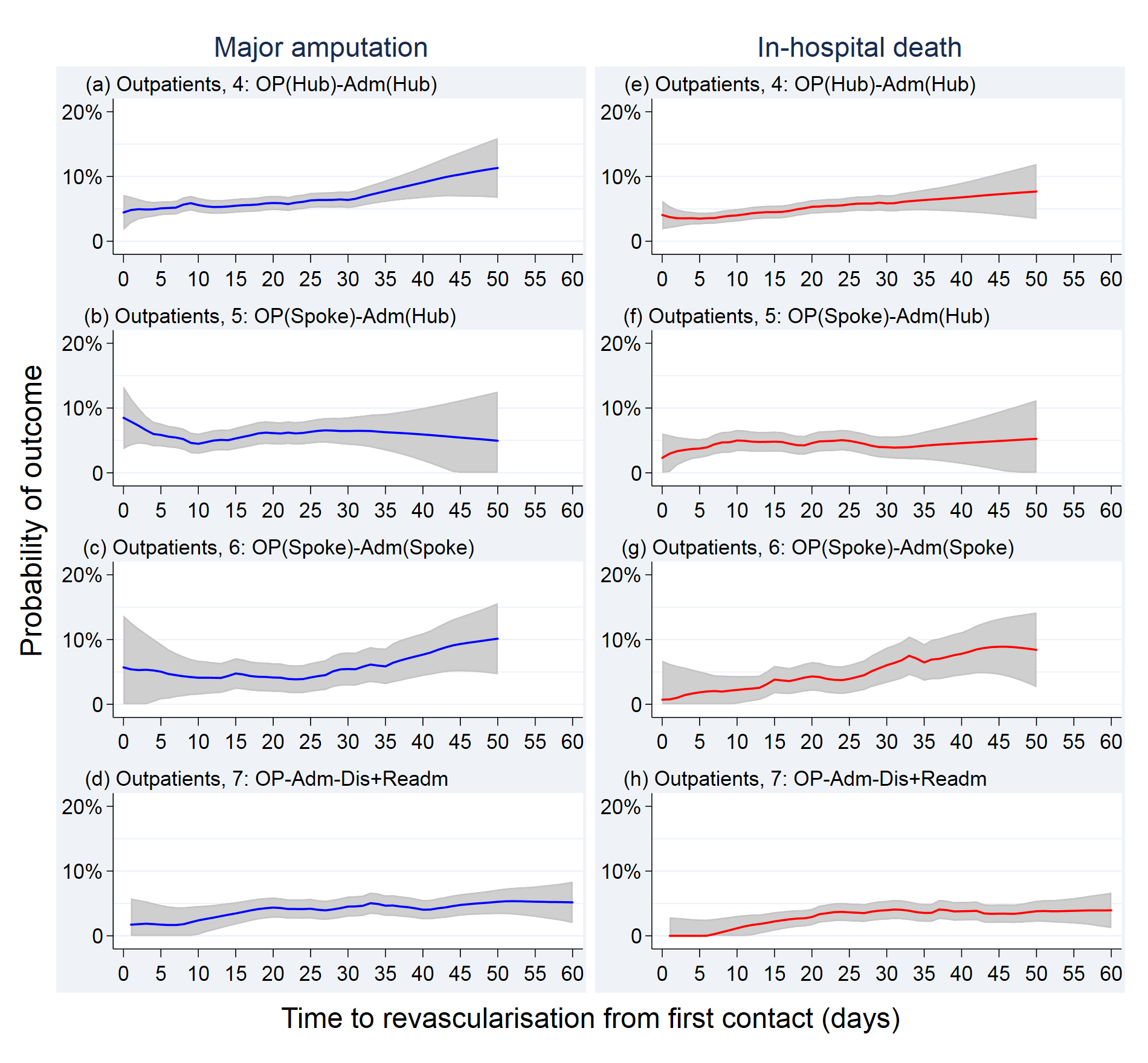

Supplement: znac109_Supplementary_Data [file znac109_supplementary_data.docx]
